# Cell Line Derived 5-FU and Irinotecan Drug-Sensitivity Profiles Evaluated in Adjuvant Colon Cancer Trial Data

**DOI:** 10.1371/journal.pone.0155123

**Published:** 2016-05-12

**Authors:** Ida Kappel Buhl, Sarah Gerster, Mauro Delorenzi, Thomas Jensen, Peter Buhl Jensen, Fred Bosman, Sabine Tejpar, Arnaud Roth, Nils Brunner, Anker Hansen, Steen Knudsen

**Affiliations:** 1 Section for Molecular Disease Biology, Faculty of Health and Medical Sciences, University of Copenhagen, Copenhagen, Denmark; 2 Medical Prognosis Institute, Hoersholm, Denmark; 3 Bioinformatics Core Facility, SIB Swiss Institute of Bioinformatics, Lausanne, Switzerland; 4 Ludwig Center for Cancer Research and Oncology Department, University of Lausanne, Lausanne, Switzerland; 5 University of Lausanne, University Institute of Pathology, Lausanne, Switzerland; 6 University Hospital Gasthuisberg, Digestive Oncology Unit, Leuven, Belgium; 7 University Hospital of Geneva, Oncosurgery Unit, Geneva, Switzerland; Baylor University Medical Center, UNITED STATES

## Abstract

**Purpose:**

This study evaluates whether gene signatures for chemosensitivity for irinotecan and 5-fluorouracil (5-FU) derived from in vitro grown cancer cell lines can predict clinical sensitivity to these drugs.

**Methods:**

To test if an irinotecan signature and a SN-38 signature could identify patients who benefitted from the addition of irinotecan to 5-FU, we used gene expression profiles based on cell lines and clinical tumor material. These profiles were applied to expression data obtained from pretreatment formalin fixed paraffin embedded (FFPE) tumor tissue from 636 stage III colon cancer patients enrolled in the PETACC-3 prospective randomized clinical trial. A 5-FU profile developed similarly was assessed by comparing the PETACC-3 cohort with a cohort of 359 stage II colon cancer patients who underwent surgery but received no adjuvant therapy.

**Results:**

There was no statistically significant association between the irinotecan or SN-38 profiles and benefit from irinotecan. The 5-FU sensitivity profile showed a statistically significant association with relapse free survival (RFS) (hazard ratio (HR) = 0.54 (0.41–0.71), p<1e-05) and overall survival (HR = 0.47 (0.34–0.63), p<1e-06) in the PETACC-3 subpopulation. The effect of the 5-FU profile remained significant in a multivariable Cox Proportional Hazards model, adjusting for several relevant clinicopathological parameters. No statistically significant effect of the 5-FU profile was observed in the untreated cohort of 359 patients (relapse free survival, p = 0.671).

**Conclusion:**

The irinotecan predictor had no predictive value. The 5-FU predictor was prognostic in stage III patients in PETACC-3 but not in stage II patients with no adjuvant therapy. This suggests a potential predictive ability of the 5-FU sensitivity profile to identify colon cancer patients who may benefit from 5-FU, however, any biomarker predicting benefit for adjuvant 5-FU must be rigorously evaluated in independent cohorts. Given differences between the two study cohorts, the present results should be further validated.

## Introduction

The antimetabolite 5-fluorouracil (5-FU) is the backbone in systemic treatment of primary and metastatic colorectal cancer (CRC), with further activity in a wide range of solid tumors including other gastrointestinal malignancies, breast cancer, head and neck cancers and ovarian carcinomas [1]. 5-FU treatment results in a survival benefit in the adjuvant setting of CRC [2] and is most often combined with oxaliplatin [3]. In treatment of metastatic CRC, 5-FU is currently combined with either the topoisomerase-1 inhibitor irinotecan as FOLFIRI regimen or with oxaliplatin as a FOLFOX regimen [4;5].

Competing variables on both tumor cell level and patient level may ensure or corrupt the efficacy of 5-FU [6] and/or irinotecan [7] and with many patients not obtaining the benefit but only the side effects of such treatment, there is an unmet need for predictive biomarkers [8]. Research on predictors of 5-FU response has mainly focused on thymidylate-synthase (TS) as a target for 5-FU and of levels of the enzyme dihydropyrimidine dehydrogenase (DPD) which metabolizes 5-FU in the liver [9–11]. Overall, conflicting results associations with treatment effect compromise the usefulness of TS and DPD as predictors for 5-FU [12;13]. Similarly, despite many attempts, no irinotecan predictive biomarkers have reached a level of evidence allowing for routine clinical use [14].

We approached the problem of predicting 5-FU or irinotecan benefit based on gene expression data by comparing associations between gene expression profiles and efficacy of the drugs in question in the National Cancer Institute US cell line repository NCI60 [15]. Models of sensitivity for these drugs were developed prior to the present study by the Medical Prognosis Institute (MPI), Denmark [16]. A second step included filtering the identified gene expression profiles against mRNA expression from a collection of 3200 human tumors. Only genes being differentially expressed in the clinical tumor material were retained in the models. An analogously constructed prediction method has recently been externally validated by biostatisticians from MD Anderson for methotrexate in acute lymphoblastic leukemia, for ABVD (doxorubicin, bleomycin, vinblastine, dacarbazine) in Hodgkin’ s lymphoma and for epirubicin in breast cancer in three separate pre-specified clinical data sets [17]. Similar models were recently tested for fulvestrant in breast cancer [18] and R-CHOP (rituximab, cyclophosphamide, doxorubuicin, vincristine, prednisone) in diffuse large B-cell lymphoma (DLBCL) [19].

In the present study we assessed a 5-FU and an irinotecan MPI sensitivity predictor. We used gene expression and clinical patient data from the PETACC-3 colon cancer (CC) patient subpopulation [20] and from a population of stage II CC patients who had undergone surgery but received no adjuvant therapy [21] profiled on the same microarray platform. In the PETACC-3 prospective randomized clinical trial, the primary objective was to investigate whether the addition of adjuvant irinotecan to adjuvant 5-FU + leucovorin (LV5FU) would improve relapse-free survival (RFS). The study concluded that adding irinotecan to LV5FU did not significantly improve survival, but they observed a statistically non-significant trend in favor of treatment with irinotecan in addition to LV5FU [20].

Conclusions from the present study were that there was no association between the irinotecan sensitivity predictor and patient outcome while the data suggest the 5-FU response profile does correlate with outcomes of patients receiving 5-FU-based adjuvant therapy. These data justify further clinical evaluation of the 5-FU response profile.

## Patients and Methods

### Predictor development based on in vitro assays

The in vitro based MPI method to develop a predictor of drug response has been described previously [16–19]. Briefly, it is an algorithm based on growth inhibition values (GI50) of the NCI60 cell lines [15] subjected to treatment with either 5-FU, irinotecan or SN-38. SN-38 is the active metabolite of irinotecan. Gene expression measurements were performed with an Affymetrix HG-U133A array. After logit normalization, genes with a Pearson’s correlation coefficient to GI50 above 0.25 or below -0.25 were considered as potential biomarkers of sensitivity and resistance to treatment and retained to contribute to the profiles for 5-FU, irinotecan or SN-38, respectively. To sort away genes only active in the in vitro setting mRNA measurements from more than 3200 snap frozen clinical tumor samples were then applied to each profile. Hereby, only markers already known to be present in patient tumors contributed to the final profiles. Each signature consists of two sets of genes (up- and down-regulated features). The profile scores were defined as the difference between the averages of the two groups of features for each of the drugs. The scores were then scaled to cover the range from 0 to 100. A sample was classified as sensitive to a treatment, if the corresponding normalized profile score was larger than 50. All other samples were considered to be resistant.

The array (Almac Diagnostics Colorectal Cancer DSA) used for analysis of mRNA in the FFPE patient samples is different from the array (HG-U133A) used for deriving the mRNA profile in vitro. That required an additional translation of the profile from one array type to another which was done by MPI before the profile was tested externally by SIB in the two study populations.

The NCI has obtained the 60 cell lines from their sources as described https://dtp.cancer.gov/default.htm [15;22].

### Data sets and arrays

For the PETACC-3 data set, the gene expression profiles were generated from FFPE colorectal cancer slides. The RNA extraction and hybridization was performed by the Almac group in two batches. Data processing is described in detail in S1 Doc. The presented results are based on 636 stage III samples.

The other cohort consisting of 359 CC stage II CC patients who had undergone surgery but received no adjuvant therapy from Kennedy et al [21] were all examined for mRNA expression similarly to the PETACC-3 samples. The cohort will hereafter be termed the Kennedy cohort.

### Statistical analysis

The current project is a result of collaboration between two independent laboratories. The profile conducted at Medical Prognosis Institute was sent to the Swiss Institute of Bioinformatics for all statistical analysis to be conducted there with a fully fixed testing procedure defined in an analysis plan prior to the execution.

Relapse-free survival (RFS) was used as main end point to compare the results on both data sets. RFS is defined as the time from the date of random allocation to the first date of relapse (local, regional, or distant), the occurrence of a second primary CC, or death. Overall survival (OS) as a secondary objective is defined as the time from the date of randomization to death of any cause.

Additional variables in the multivariable analyses were treatment group, age, gender, tumor site, T stage (T1-2, T3, and T4), N stage (N1 and N2) and tumor grade (G1-2 and G3-4), KRAS mutation, BRAF mutation and microsatellite instability (MSI) when the variables were available.

The analysis plan was designed to look for associations between prediction scores and clinical outcome. Patients were discriminated in 2 groups based on the computed profile score. Patients with a 5-FU profile score smaller or equal to 50 were labeled as poor prognosis (resistant). The other patients were considered as good prognosis (sensitive) in relation to 5-FU treatment.

A log-rank test was used to test the profile as a discrete variable comparing the survival curves of patients predicted sensitive to those predicted resistant. Second, the plan was to fit (multivariable) Cox Proportional Hazards models using the continuous profile scores.

Further, the hazard ratios for the 5-FU profile for relapse-free survival in the PETACC-3 and the Kennedy cohort were compared using a z-test. This is based on the assumptions that with the null hypotheses that the two HR are equal, that the error distribution of the estimates are normally distributed and that the two populations are comparable. However, it must be stressed that the two populations represent two distinct cohorts with all the differences this can lead to.

## Results

### Expression arrays and cell-line sensitivity data

The irinotecan profile derived from the NCI60 cell lines consisted of 38 positively correlated genes and 32 negatively correlated genes. The genes were annotated to 353 positively correlated and 166 negatively correlated ALMAC probe sets from the ALMAC Colorectal Cancer DSA array. The 5-FU profile consisted of 91 positively correlated genes and 114 negatively correlated genes. The probe sets from Affymetrix were mapped to 232 positively correlated and 437 negatively correlated probe sets from the ALMAC Colorectal Cancer DSA array.

The full list of probe sets and gene names for the irinotecan predictor appears from S1 Table; for the 5-FU predictor it is available as S2 Table.

### Baseline patient characteristics

From the original PETACC-3 patient cohort, we identified a subgroup of 636 stage III CC patients with available high quality mRNA expression data. This subgroup was used to test the prognostic and predictive significance of the MPI irinotecan, SN-38 and 5-FU profiles, respectively. Since the irinotecan and SN-38 data were almost identical we here only report on the irinotecan results. Baseline patient characteristics for the PETACC-3 study population and the presently investigated subpopulation are reported in S3 Table parts A and B. It is seen that the subpopulation was representative of the total stage III PETACC-3 study population. Furthermore, this subpopulation did not show major differences to the main PETACC-3 cohort regarding benefit from adjuvant chemotherapy. Baseline patient characteristics for the Kennedy cohort are presented in S3 Table part C.

### Association between irinotecan profile and baseline characteristics

S4 Table part A shows associations between the irinotecan predictive profile and several baseline clinicopathological characteristics for the PETACC-3 subpopulation. We noticed a significant association between the MSI/MSS status and the profile score in the subpopulation of the PETACC-3 study. This was not taken into account for the subsequent analysis.

### The irinotecan gene expression profile and patient prognosis

S4 Table part B shows that there was no statistically significant effect of the irinotecan score on RFS (HR = 0.93; 95% CI = (0.77, 1.12); P = 0.450, N = 558) when tested in the total subgroup. The results for OS are given in S4 Table part C.

In order to test for a potential interaction between the profile score and the treatment group, we fitted models including an interaction term for RFS and for OS (outcome ~ treatment_group + profile_score + treatment_group:profile_score). For the irinotecan profile, none of the three terms led to a HR statistically significantly different from 1 (for OS and RFS, se S4 Table part D).

Kaplan Meier survival curves for the irinotecan profile are presented as S1 Fig. This profile score was not associated with a difference in survival neither for RFS (Panel A, HR = 1.07; 95% CI = (0.81, 1.41); P = 0.65; N = 636) nor OS (Panel B, HR = 1.14; 95% CI = (0.82, 1.57); P = 0.43; N = 636)).

### Subgroup analyses

A significant separation of groups in favor of a high sensitivity score was neither obtained when comparing the irinotecan profile with RFS in the “5-FU only” (no irinotecan added) treatment group (HR = 0.89; 95% CI = (0.6, 1.31); P = 0.55; N = 307), with RFS in FOLFIRI treatment group (HR = 1.28; 95% CI = (0.86, 1.91); P = 0.23; N = 329), with OS in “5-FU only” treatment group (HR = 0.83; 95% CI = (0.53, 1.3); P = 0.41; N = 307) nor with OS in FOLFIRI treatment group (HR = 1.58; 95% CI = (1, 2.5); P = 0.05; N = 329); see S1 Fig panel C, D and E, F, respectively.

### Association between 5-FU profile and baseline characteristics

S5 Table part A shows associations between the 5-FU predictive profile and several baseline clinicopathological characteristics for the PETACC-3 subpopulation. The table showing the same information for the samples from the Kennedy cohort is available as S6 Table part A. It is seen that T-stage has a significant association with the 5-FU profile in the PETACC-3 subpopulation in the simple and in the multiple regression models. However, the effect is only statistically significant when comparing T1-2 (smallest group) to T3 (largest group). This was not taken into consideration for the subsequent analysis. No particularities were noticed in the Kennedy cohort. We do not have MSI/MSS information for the Kennedy cohort.

### The 5-FU gene expression profile and patient prognosis

Table 1 and S5 Table part B display the results from the fitted (multivariable) Cox Proportional Hazards models for the PETACC-3 subpopulation. Table 1 shows that the effect of the 5-FU profile on outcome (RFS) was statistically significant even when correcting for a set of standard clinicopathological variables (HR = 0.72, 95% CI = (0.62, 0.84), P = 0,00002, N = 558), with the high scoring (sensitive) group having longer RFS.

**Table 1:** Association between RFS and the 5-FU profile score in the subpopulation of the PETACC-3 study.

	HR_multi	CI_multi	pval_multi	HR_sing	CI_sing	pval_sing
FU5Pred (IQR scaled)	0.72	(0.62, 0.84)	0.00002	0.69	(0.6, 0.79)	0.00000
trt_grp (FOLFIRI vs 5-FU/FA)	0.87	(0.66, 1.14)	0.32012	0.87	(0.68, 1.12)	0.29253
age (in years)	1.00	(0.99, 1.01)	0.86710	1.00	(0.99, 1.01)	0.73414
sex (female vs male)	1.04	(0.78, 1.39)	0.76737	0.91	(0.7, 1.18)	0.46790
site (right vs left)	1.15	(0.86, 1.55)	0.34834	1.10	(0.85, 1.43)	0.47551
tstage (T12 vs T3)	0.60	(0.3, 1.19)	0.14288	0.41	(0.21, 0.8)	0.00894
tstage (T4 vs T3)	1.72	(1.23, 2.39)	0.00131	1.86	(1.38, 2.52)	0.00005
nstage (N2 vs N1)	1.98	(1.49, 2.63)	0.00000	2.21	(1.71, 2.85)	0.00000
grade (G-34 vs G-12)	1.57	(0.99, 2.49)	0.05639	1.57	(1.09, 2.27)	0.01570
BRAF (mut vs wt)	1.20	(0.68, 2.11)	0.53461	1.16	(0.7, 1.94)	0.55788
KRAS (mut vs wt)	1.51	(1.12, 2.03)	0.00620	1.29	(0.99, 1.68)	0.05835
MSI (MSI-H vs MSS)	0.46	(0.25, 0.82)	0.00835	0.60	(0.35, 1.03)	0.06384

The first three columns relate to results from a multivariable Cox Proportional Hazards model. The last three columns relate to the results of each variable being tested in a simple (single explanatory variable) Cox Proportional Hazards model. The 5-FU profile score is statistically significantly associated with RFS, even when correcting for other covariates.

Full model: n = 558, number of events = 212, 78 observations deleted due to missingness.

As with the irinotecan profile in order to test for a potential interaction between the profile score and the treatment group, we fitted models including an interaction term for RFS and for OS (outcome ~ treatment_group + profile_score + treatment_group:profile_score). For the 5-FU profile, only the HR for the profile score was statistically significantly different from 1 (for OS and RFS, see S5 Table part C for details). The treatment group and the interaction term did not have an HR statistically significantly different from 1.

**Fig 1:**
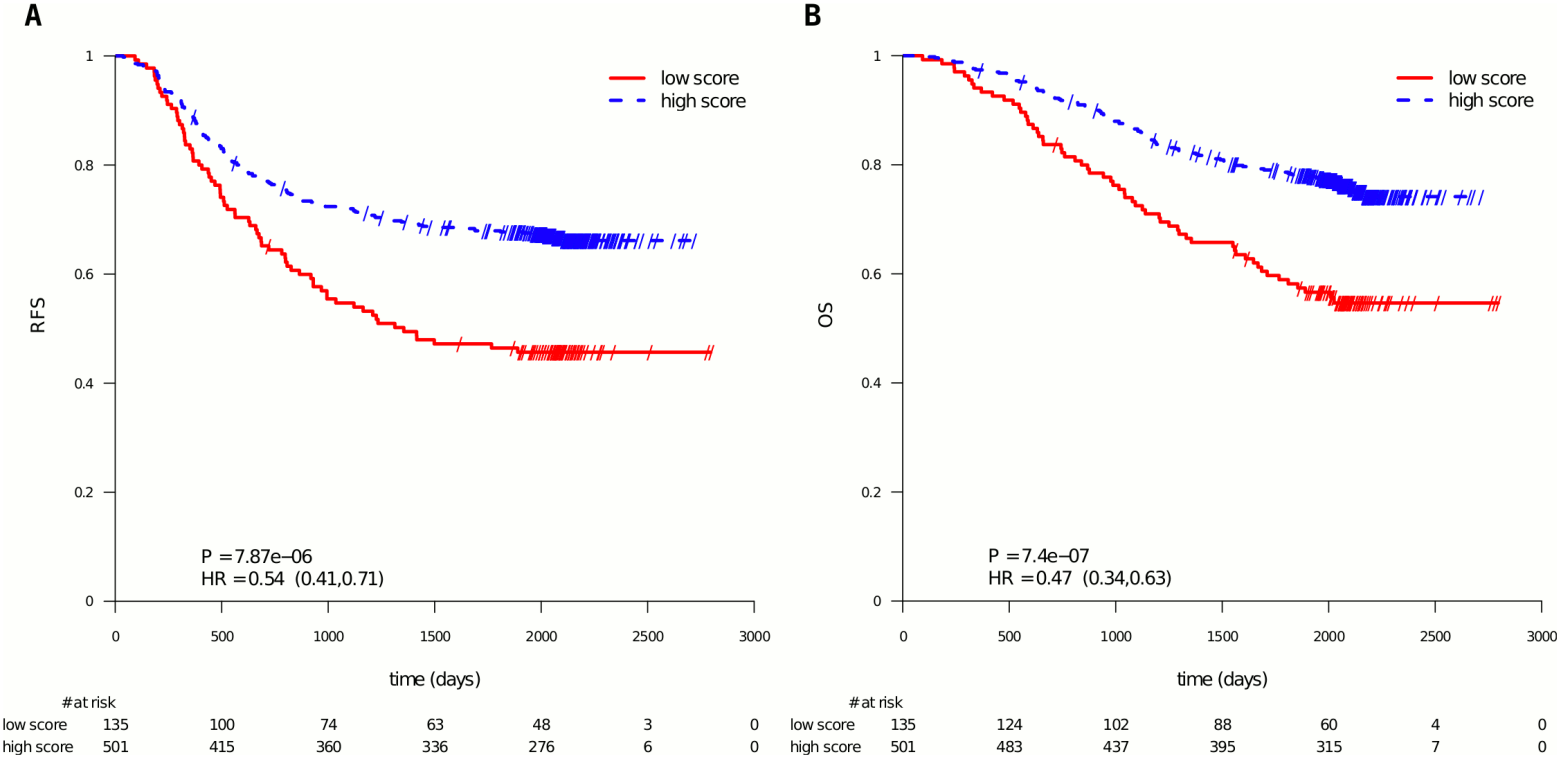
PETACC-3 subpopulation of patients stratified by the 5-FU profile score. Patients with a 5-FU profile score smaller or equal to 50 were labeled as poor prognosis, the other ones as good prognosis in relation to 5-FU treatment. 5-FU was included in all patients' treatment. The curves show that the 5-FU profile significantly separates the patients into good and poor outcome (using RFS as endpoint in panel A, OS in panel B).

In Fig 1 Kaplan Meier curves are shown for the endpoint RFS (panel A) and OS (panel B). In each panel the patients are stratified according to their 5-FU profile score. It is seen that the score splits the patients into a good and a poor prognosis group. Patients classified as sensitive had a statistically significantly better survival with RFS (HR = 0.54; 95% CI = (0.41, 0.71); P = 7.87e-6; N = 636; based on dichotomized profile scores) and OS (HR = 0.47; 95% CI = (0.34, 0.63), P = 7.4e−7; based on dichotomized profile scores), (see method section).

### Subgroup analyses

**Fig 2:**
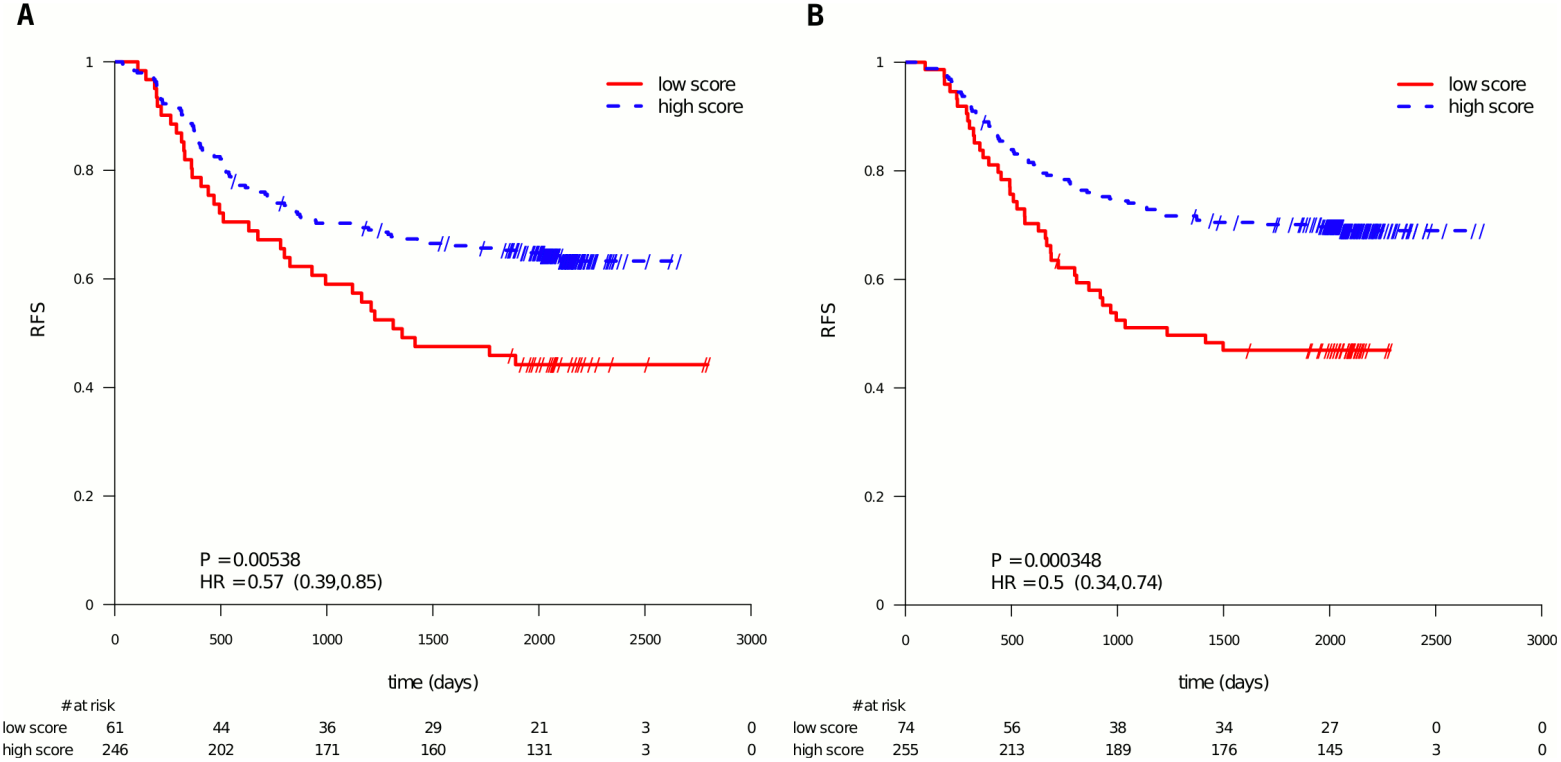
PETACC-3 subpopulation of patients stratified by the 5-FU profile score, RFS. Same data as in Fig 1A, but presenting the results per treatment arm. Panel A shows the data of the patients in the 5-FU/FA arm, Panel B the data for the patients in the FOLFIRI treatment arm. The used endpoint is RFS.

**Fig 3:**
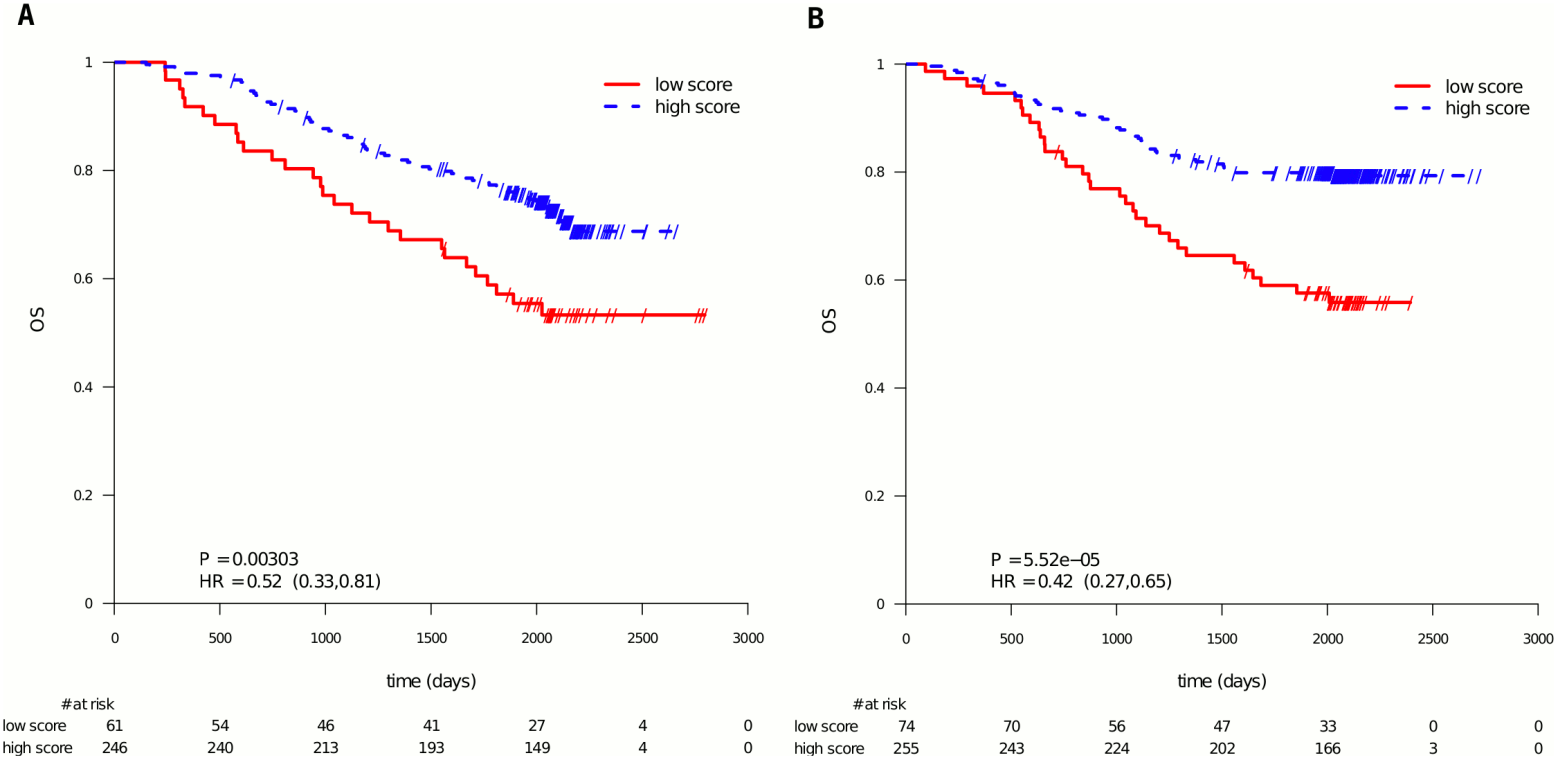
PETACC-3 subpopulation of patients stratified by the 5-FU profile score, OS. Same data as in Fig 1B, but presenting the results per treatment arm. Panel A shows the data of the patients in the 5-FU/FA arm, Panel B the data for the patients in the FOLFIRI treatment arm. The used endpoint is OS.

The panels A in Figs 2 (RFS) and 3 (OS) display the data from patients in the “5-FU only” (no irinotecan added) treatment group. It is seen that the 5-FU profile also significantly splitted the patients in this treatment arm into a good and a poor prognosis subgroup with RFS in Fig 2 (HR = 0.57, 95% CI = (0.39, 0.85), P = 5.38e-3, N = 307) and OS in Fig 3 (HR = 0.52; 95% CI = (0.33, 0.81); P = 3.03e-3; N = 307).

The same analysis was performed on the FOLFIRI treated patients (see panels B in Figs 2 and 3). It is seen that the 5-FU profile also splitted these patients into two significantly different prognostic groups for RFS (HR = 0.50, 95% CI = (0.34, 0.74), P = 3.48e-4, N = 329) and OS (HR = 0.42; 95% CI = (0.27, 0.65); P = 5.52e-5; N = 329).

**Fig 4:**
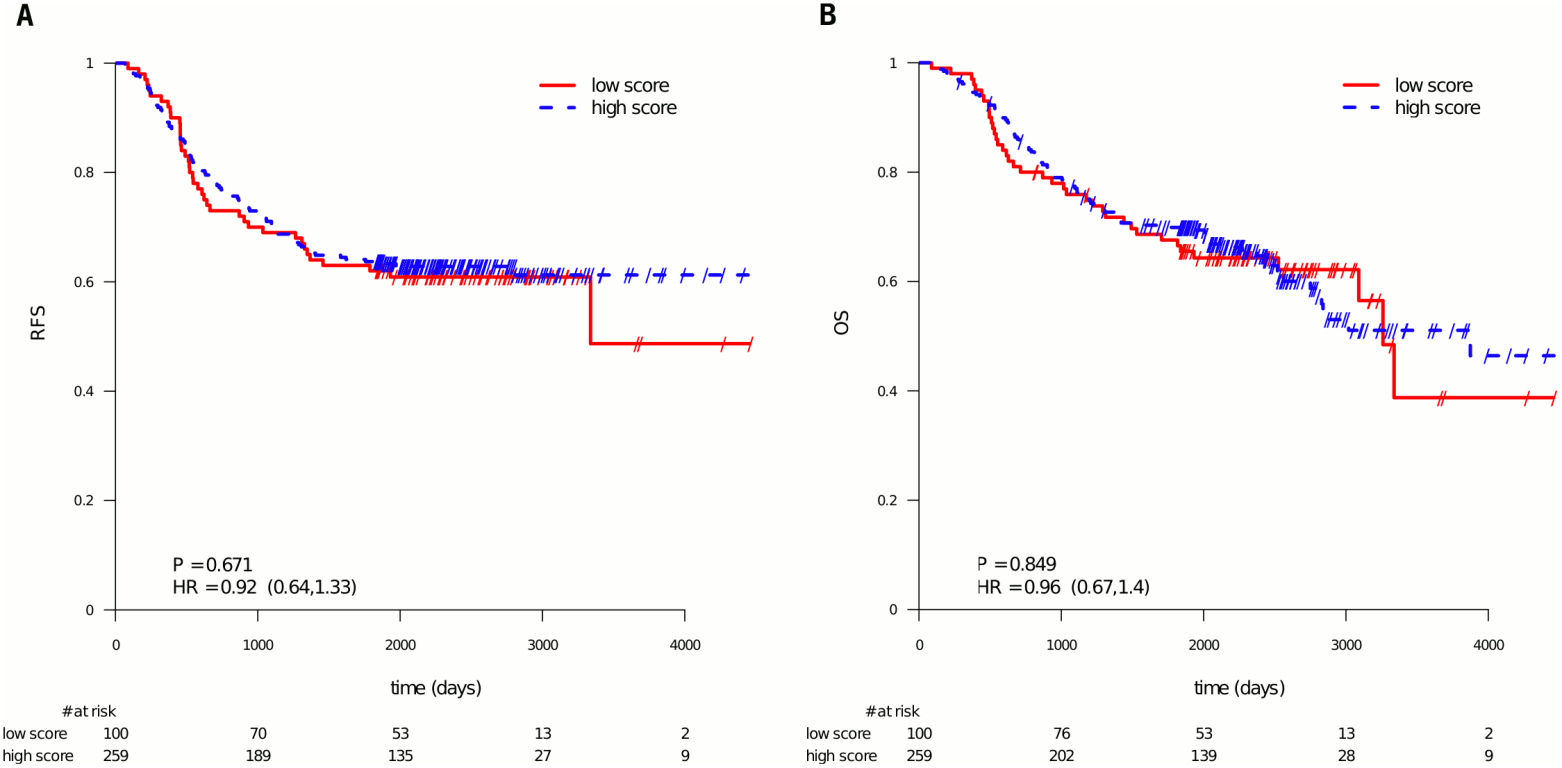
Patients from the Kennedy cohort stratified by the 5-FU profile score. Patients with a 5-FU profile score smaller or equal to 50 were labeled as low score, the other ones as high score in relation to 5-FU treatment. The curves show that the 5-FU profile does not separate the CC patients into good and poor outcome (using RFS as endpoint in panel A, OS in panel B).

Based on the above results showing a significant prognostic value of the 5-FU expression profile in patients enrolled in the PETACC-3 study in which all patients received 5-FU, we then applied the 5-FU profile to a cohort of CC patients who had no adjuvant therapy to surgery, the Kennedy cohort. In the Kennedy cohort [21] no statistically significant differences (univariate and multivariate analysis) in RFS (HR = 0.92; 95% CI = (0.64, 1.33); P = 0.671, N = 359) or OS (HR = 0.96, 95% CI = (0.67, 1.4); P = 0.849; N = 359) were observed when the population was dichotomized by the 5-FU predictive profile, see Fig 4. It was further checked that also performing an analysis using the continuous profile score did not lead to statistically significant differences in RFS (S6 Table part B) or OS (S6 Table part C).

We then asked if there was a significant difference in the hazard ratios for the 5-FU profile for RFS in both treatment arms (5-FU and FOLFIRI) of the PETACC-3 (HR = 0.54; 95% CI = (0.41, 0.71)) and for the Kennedy cohort (HR = 0.92; 95% CI = (0.64, 1.33)). A test comparing the two hazard ratios demonstrated a significant difference (z-test, p = 0.02, see methods).

## Discussion

In this study gene expression response profiles for irinotecan and 5-FU developed based on the NCI60 cell line repository and filtered using clinical tumor material representing different tumor types to exclude cell-line specific genes [18] were evaluated retrospectively in tumor material obtained from the PETACC-3 prospective and randomized clinical trial [20] and in publically available data from stage II CC patients, the Kennedy cohort [21].

MPI’s method uses the correlation between sensitivity to a drug and gene expression in a panel of cell lines. The resulting gene expression profile of genes can explain the differential sensitivity in vitro. But this gene expression profile is not directly predictive in a clinical setting. To predict patient responses to the same drug, a translation from the in vitro profile to a clinical profile is necessary. MPI filters the in vitro profile through a systems biological network derived from analysis of patient samples.

For application to the clinical studies, the genes found to be markedly up- or down-regulated in the profiles were translated by the MPI laboratory to corresponding ALMAC probe sets in order to be tested in the two clinical data sets.

Using a prospective-retrospective trial design is considered necessary to reach a sufficient level of evidence to determine clinical utility when validating predictive biomarkers [23]. The design of the PETACC-3 trial allows for a direct validation of irinotecan predictive biomarkers while it lacks an untreated arm to test biomarker candidates to predict benefit from 5-FU treatment. A total of 636 stage III CC patients from the PETACC-3 study were included in the present study.

The most interesting finding of the present study was that the 5-FU response profile significantly separated the PETACC-3 patients into a larger group (79% of the patients) of good and a smaller group (21% of the patients) of poor prognosis independently from the known prognostic clinicopathological parameters TN stage, MSI/MSS-status, site, grade, and activating mutations in KRAS and BRAF. In contrast, the irinotecan expression profile did not lead to a statistically significant split. When dividing the present patient cohort according to treatment arm, similar results were obtained, meaning that the irinotecan profile did not provide predictive nor prognostic information.

The scaling of the profile was a linear transformation that does not affect the distribution but only the interpretability of the score. The cutoff of 50 is close to the median in many populations, but in the stage III PETACC population it was far from the median. We had to stick to the cutoff of 50, however, because this was defined in the statistical analysis plan before looking at the clinical data.

Since patients in both treatment groups of the PETACC-3 trial received 5-FU, it is not possible to distinguish a profile indicative of smaller intrinsic risk from a profile predictive of a survival benefit due to 5-FU efficacy. A purely prognostic profile should recognize different risk groups also in untreated populations. However, we did not observe any statistically significant prognostic effect in the Kennedy cohort, not even a trend. This could be interpreted as the difference between good and poor survival groups in the PETACC-3 cohort as identified by the 5-FU profile, is mainly due to difference in benefit from 5-FU.

An alternative interpretation is that the profile has a strong purely prognostic value in stage III (PETACC-3) but not in stage II (Kennedy), or that the observation is due to other and unknown differences between the two patient cohorts cannot be excluded.

A comparison of biomarker validation results from multiple data sets has several limitations. In this study, first, the PETACC-3 subpopulation consisted of stage III CC patients, the Kennedy cohort of stage II CC patients. Second, the set of available covariates was different and some important characteristics, such as MSI status, were not always available. In the PETACC-3 subpopulation there is an overrepresentation of male patients while in the Kennedy cohort this is better balanced. Furthermore, the balance of left- versus right-sided tumors in the PETACC-3 cohort and the Kennedy cohort differ (with many missing data on primary tumor location for the patients of the Kennedy cohort).

It should be noted that finding suitable data sets to assess a potential predictive ability of a biomarker for adjuvant 5-FU and/or irinotecan treatment in CC patients is difficult. Most studies comparing treatment with 5-FU to untreated patients were conducted many years ago and the patient populations would not be representative of today’s CC patients anymore. For example, among many improvements, surgical procedures have been significantly further developed.

Despite the two cohorts being different, we asked if there was a significant difference in the hazard ratios for the 5-FU profile for RFS in PETACC-3 (HR = 0.54; 95% CI = (0.41, 0.71)) and in the Kennedy cohort (HR = 0.92; 95% CI = (0.64, 1.33)) with a test comparing the two hazard ratios which demonstrated a significant difference (z-test, p = 0.02). However, due to major differences between the two analyzed cohorts, this result needs further clinical validation in tumor material obtained from an appropriately designed clinical study.

The irinotecan profile failed to demonstrate any significant separation of the PETACC-3 patients or the Kennedy cohort. One explanation to this finding is that, since there was no significant effect of adding treatment with irinotecan in the original PETACC-3 study, any subpopulation that would benefit from adding irinotecan might be too small to be detected with the available patient samples. Another explanation that cannot be excluded is that there is no benefit from adjuvant irinotecan treatment in stage III CC. This latter explanation is supported by the CALGB 89803 study which had a similar design as the PETACC-3 study and which also failed to demonstrate any significant benefit from addition of irinotecan to LV5FU [24].

Adjuvant treatment of CC patients in stage II or III has stagnated even with many attempts of introducing new drugs during the past decade [25]. Biomarkers to assist the physician in regards of prognosis, of prediction of treatment efficacy and expected severe toxicities to antineoplastic treatment of CC have long been eagerly awaited. Only few prognostic biomarkers have been implemented clinically and no biomarkers for 5-FU or irinotecan sensitivity have been developed to a stage where they can be used in routine treatment of CC. An important issue with the validation of biomarkers in adjuvant treatment of CC is that patients not experiencing a disease recurrence could either have been cured by the primary surgery (representing sensitive or resistant tumors) or by the subsequent chemotherapy (representing sensitive tumors), while patients having disease recurrence are considered to have resistant tumors. This is an inherent problem to which there is no satisfying solution when analyzing predictive biomarkers in the adjuvant setting.

In conclusion, the present study presents a novel 5-FU mRNA expression profile which could separate the stage III subpopulation of the PETACC-3 patient cohort into subgroups with statistically significant different outcome. By also applying this profile to a cohort of untreated CC patients, we here provide suggestions for a possible association between the 5-FU profile and benefit from adjuvant 5-FU treatment in stage III CC patients. We are currently seeking to validate our 5-FU data in an independent patient cohort.

## Supporting Information

S1 Doc Data Processing in PETACC-3 data. The document describes human sample material and data processing with R statistical software on the PETACC-3 data.
(DOCX)

S1 Fig PETACC-3 subpopulation of patients stratified by their irinotecan profile score. Patients with an irinotecan profile score smaller or equal to 50 were labeled as poor prognosis, the other ones as good prognosis in relation to 5-FU treatment. The curves show that the irinotecan profile does not split the patients into good and poor outcome (using RFS as endpoint in panel A, OS in panel B). Panels C and D show the same data as in panel A, but presenting the results per treatment arm. Panel C shows the data of the patients in the 5-FU/FA arm, Panel D the data for the patients in the FOLFIRI treatment arm. The used endpoint is RFS. Panels E and F show the same data as in panel B, but presenting the results per treatment arm. Panel E shows the data of the patients in the 5-FU/FA arm, Panel F the data for the patients in the FOLFIRI treatment arm. The used endpoint is OS.
(PDF)

S1 Table Probe sets in the irinotecan profile. **Part A. Probe sets for genes overexpressed in cell lines sensitive to irinotecan.** The table shows the Almac probe sets that corresponds to the genes identified in cell lines. Note that some genes have several matching probe sets, the mapping is neither unique nor inambiguous. **Part B. Probe sets for genes overexpressed in cell lines resistant to irinotecan.** The table shows the Almac probe sets that corresponds to the genes identified in cell lines. Note that some genes have several matching probe sets, the mapping is neither unique nor inambiguous.
(PDF)

S2 Table Probe sets in the 5-FU profile. **Part A. Probe sets for genes overexpressed in cell lines sensitive to 5-FU.** The table shows the Almac probe sets that corresponds to the genes identified in cell lines. Note that some genes have several matching probe sets, the mapping is neither unique nor inambiguous. **Part B. Probe sets for genes overexpressed in cell lines resistant to 5-FU.** The table shows the Almac probe sets that corresponds to the genes identified in cell lines. Note that some genes have several matching probe sets, the mapping is neither unique nor unambiguous.
(PDF)

S3 Table Baseline demographics. **Part A. Baseline demographics for the patients from the PETACC-3 study.** The table provides an overview of the distribution of all patients in the PETACC-3 study according to the major clinicopathological variables. Some variables were only assessed in the profiled samples. This information is provided in italics. For example, the KRAS mutation status was not assessed in most of the samples (1395 missing values). Hence, the information that only 24% of the samples are wild-type is somewhat misleading: In italics, one can see that among the assessed samples, 60.4% were found to be wild-type and 39.4% to be KRAS mutated. **Part B. Baseline demographics for the subset of stage III patients from the PETACC-3 study selected for the present analysis.** The table provides an overview of the distribution of the PETACC-3 patients used in the present analysis according to the major clinicopathological variables. The information provided in italics shows the actual percentages among all samples without missing values. For example, the KRAS mutation status was not assessed in 40 samples (6.3% of the patients). Hence, the information that only 55.7% of the samples are wild-type can be misleading. In italics, one can see that actually 59.4% of the assessed samples were found to be wild-type and 40.6% to be KRAS mutated. **Part C. Baseline demographics for the stage II CC patients from Kennedy et al [21].** The table provides an overview of the distribution of all (stage II) patients in the study published in Kennedy et al [21] according to the major clinicopathological variables.
(PDF)

S4 Table Irinotecan profile. **Part A. Association between the irinotecan profile score and clinicopathological parameters for the PETACC-3 subpopulation.** We tested for association between the irinotecan profile score and the major clinicopathological parameters in the clinical data. It is seen that site, grade and MSI has a significant association with the irinotecan profile score in the PETACC-3 subpopulation in the simple and in the multiple regression models. Detailed results are provided in the table below. The first two columns relate to the results from a multivariable regression model. The last two columns relate to the results of each variable being tested in a simple (single explanatory variable) model. The estimates for the intercepts are not reported. **Part B. Association between RFS and the irinotecan profile score in the subpopulation of the PETACC-3 study.** The first three columns relate to results from a multivariable Cox Proportional Hazards model. The last three columns to the results of each variable was tested in a simple (single explanatory variable) Cox Proportional Hazards model. The irinotecan profile score was not statistically significantly associated with RFS. The estimates for the other variables are in line with results obtained in former analyses on the PETACC-3 data. **Part C. Association between OS and the irinotecan profile score in the subpopulation of the PETACC-3 study.** The first three columns relate to results from a multivariable Cox Proportional Hazards model. The last three columns to the results of each variable was tested in a simple (single explanatory variable) Cox Proportional Hazards model. The irinotecan profile score was not statistically significantly associated with OS. **Part D. Interaction between the irinotecan profile score and the treatment group, RFS and OS.** In order to test for a potential interaction between the profile score and the treatment group, we fitted models for RFS and for OS including an interaction term (outcome ~ treatment_group + profile_score + treatment_group:profile_score). For the irinotecan profile, none of the three terms in the model showed a HR statistically significantly different from 1.
(PDF)

S5 Table 5-FU profile in PETACC-3. **Part A. Association between the 5-FU profile score and clinicopathological parameters for the PETACC-3 subpopulation.** We tested for association between the 5-FU profile score and the major clinicopathological parameters in the clinical data. It is seen that T stage has a significant association with the 5-FU profile in the PETACC-3 subpopulation in the simple and in the multiple regression models. However, the effect is only statistically significant when comparing T1-2 (smallest group) to T3 (largest group). Detailed results are provided in the table below. The first two columns relate to the results from a multivariable regression model. The last two columns relate to the results of each variable being tested in a simple (single explanatory variable) model. The estimates for the intercepts are not reported. **Part B. Association between OS and the 5-FU profile score in the subpopulation of the PETACC-3 study.** The first three columns relate to results from a multivariable Cox Proportional Hazards model. The last three columns relate to the results of each variable being tested in a simple (single explanatory variable) Cox Proportional Hazards model. The 5-FU profile score is statistically significantly associated with OS, even when correcting for other covariates. **Part C. Interaction between the 5-FU profile score and the treatment group, RFS and OS.** In order to test for a potential interaction between the profile score and the treatment group, we fitted models for RFS and for OS including an interaction term (outcome ~ treatment_group + profile_score + treatment_group:profile_score). For the 5-FU profile, only the HR of the profile score was statistically significantly different from 1 after adjustment for the other explanatory variables in the model. The treatment group and the interaction term did not have a HR statistically significantly different from 1.
(PDF)

S6 Table 5-FU profile in the Kennedy cohort. **Part A. Association between the 5-FU profile score and clinicopathological parameters for the samples from the Kennedy cohort.** The first two columns relate to the results from a multivariable regression model. The last two columns relate to the results of each variable being tested in a simple (single explanatory variable) model. The estimates for the intercepts are not reported in the table below. The only statistically significant association found is with 'age'. **Part B. Association between relapse free survival and the 5-FU profile score in the Kennedy cohort.** The first three columns relate to results from a multivariable Cox Proportional Hazards model. The last three columns relate to the results of each variable being tested in a simple (single explanatory variable) Cox Proportional Hazards model. The 5-FU profile score was not statistically significantly associated with RFS. **Part C. Association between overall survival and the 5-FU profile score in the Kennedy cohort.** The first three columns relate to results from a multivariable Cox Proportional Hazards model. The last three columns relate to the results of each variable being tested in a simple (single explanatory variable) Cox Proportional Hazards model. The 5-FU profile score was not statistically significantly associated with OS.
(PDF)
