# Supplementary material for: Cell Line Derived 5-FU and Irinotecan Drug-Sensitivity Profiles Evaluated in Adjuvant Colon Cancer Trial Data
Source: PLoS One. 2016 May 12;11(5):e0155123. doi: 10.1371/journal.pone.0155123 (PMC4865183; doi:10.1371/journal.pone.0155123)
Supplement: S1 Doc — The document describes human sample material and data processing with R statistical software on the PETACC-3 data. (DOCX) [file pone.0155123.s001.docx]

**S1 Doc**

Gene expression profiles were generated from FFPE colorectal cancer slides of

the PETACC-3 trial cohort, a set of diverticulosis and normal tissue specimens

from the University of Lausanne. The RNA extraction and hybridization was

performed by the Almac group in two batches. Data processing was performed

using R statistical software as follows. For quality control, NUSE (Normalized

Unscaled Standard Error) values were computed by batch with the Bioconductor

package affyPLM (functions fitPLM and NUSE) and we confirmed that the

distributions were similar for the two batches10. Only the samples with median

NUSE <= 1.04 were retained and normalized by batch using the rmaPLM function

with default settings (background correction, quantile normalization and

median polish probe summarization). Batch effect removal was done with the R

function ComBat in the sva package, with parameters trained on 47 bridging

tumor samples profiled in both batches and then applied to all samples. Batch

was the only covariate in this model. We then adjusted to alleviate an

observed technical effect between NUSE and intensities: ComBat was applied

with eight equally sized NUSE-bins as batch variable. Only one sample for each

bridging pair was kept (the one in the first batch). Normal and diverticulosis

samples as well as stage II samples were discarded in this study. Thus, the

presented results are based on 636 stage III samples.
