# Supplementary figures and images for: Cell Line Derived 5-FU and Irinotecan Drug-Sensitivity Profiles Evaluated in Adjuvant Colon Cancer Trial Data
Source: PLoS One. 2016 May 12;11(5):e0155123. doi: 10.1371/journal.pone.0155123 (PMC4865183; doi:10.1371/journal.pone.0155123)

S1 Fig

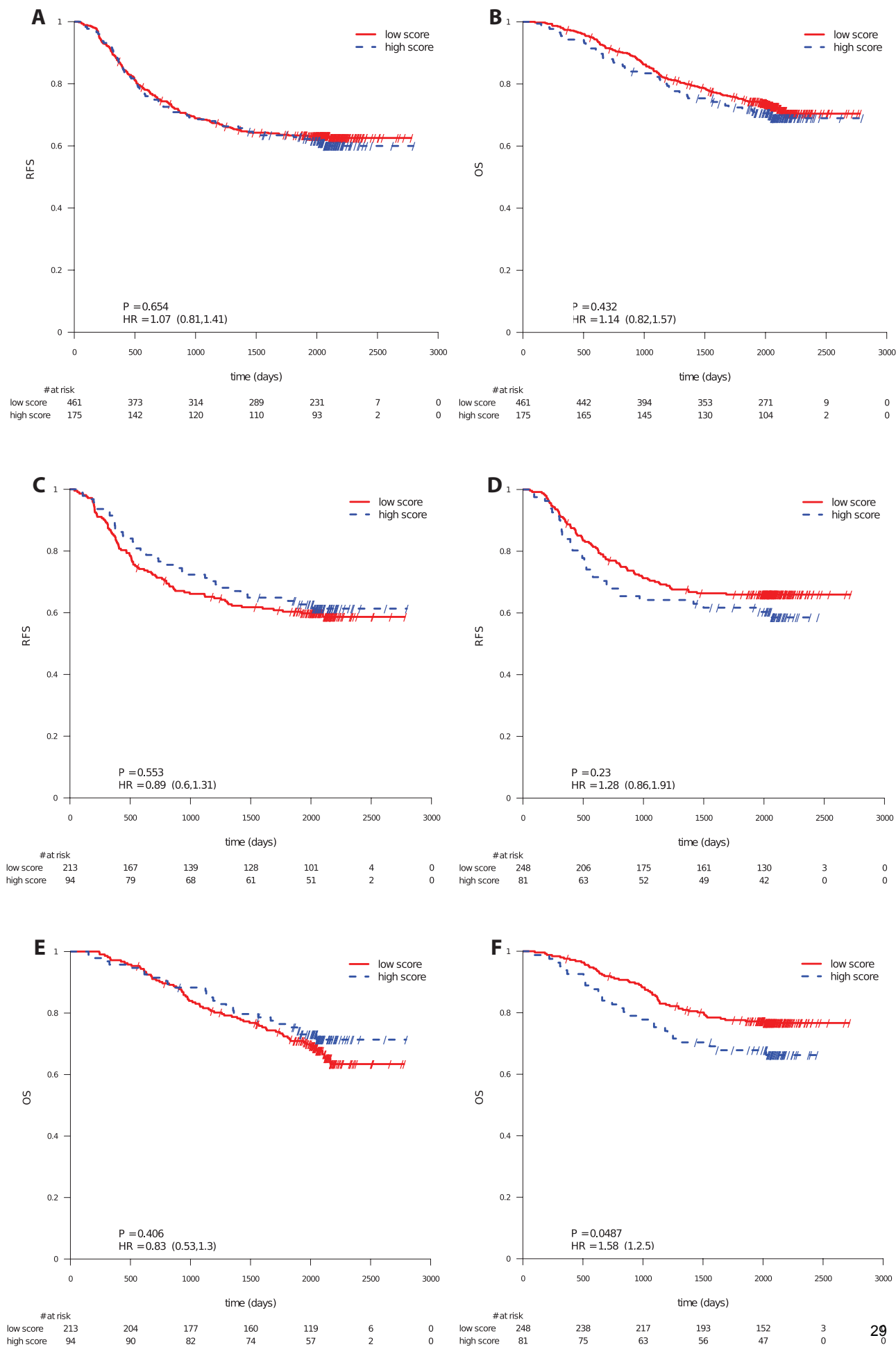

Supplement: S1 Fig — Patients with an irinotecan profile score smaller or equal to 50 were labeled as poor prognosis, the other ones as good prognosis in relation to 5-FU treatment. The curves show that the irinotecan profile does not split the patients into good and poor outcome (using RFS as endpoint in panel A, OS in panel B). Panels C and D show the same data as in panel A, but presenting the results per treatment arm. Panel C shows the data of the patients in the 5-FU/FA arm, Panel D the data for the patients in the FOLFIRI treatment arm. The used endpoint is RFS. Panels E and F show the same data as in panel B, but presenting the results per treatment arm. Panel E shows the data of the patients in the 5-FU/FA arm, Panel F the data for the patients in the FOLFIRI treatment arm. The used endpoint is OS. (PDF) [file pone.0155123.s002.pdf]
