# Supplementary material for: Cell Line Derived 5-FU and Irinotecan Drug-Sensitivity Profiles Evaluated in Adjuvant Colon Cancer Trial Data
Source: PLoS One. 2016 May 12;11(5):e0155123. doi: 10.1371/journal.pone.0155123 (PMC4865183; doi:10.1371/journal.pone.0155123)
Supplement: S1 Table — Part A. Probe sets for genes overexpressed in cell lines sensitive to irinotecan. The table shows the Almac probe sets that corresponds to the genes identified in cell lines. Note that some genes have several matching probe sets, the mapping is neither unique nor inambiguous. Part B. Probe sets for genes overexpressed in cell lines resistant to irinotecan. The table shows the Almac probe sets that corresponds to the genes identified in cell lines. Note that some genes have several matching probe sets, the mapping is neither unique nor inambiguous. (PDF) [file pone.0155123.s003.pdf]

S1 Table part A

probeset_id	entrez_id	hgnc_symbol	Description
200016_x_at	3178	HNRNPA1	Heterogeneous nuclear ribonucleoprotein A1
ADXCRAG_AB041046_s_at	29109	FHOD1	Formin homology 2 domain containing 1
ADXCRAG_AB050477_s_at	116496	FAM129A	Family with sequence similarity 129, member A
ADXCRAG_AB066567_s_at	467	ATF3	Activating transcription factor 3
ADXCRAG_AF016370_s_at	9129	PRPF3	PRP3 pre-mRNA processing factor 3 homolog (S. cerevisiae)
ADXCRAG_AF071593_s_at	7468	WHSC1	Wolf-Hirschhorn syndrome candidate 1
ADXCRAG_AF071594_s_at	7468	WHSC1	Wolf-Hirschhorn syndrome candidate 1
ADXCRAG_AF083391_at	7468	WHSC1	Wolf-Hirschhorn syndrome candidate 1
ADXCRAG_AF083391_s_at	7468	WHSC1	Wolf-Hirschhorn syndrome candidate 1
ADXCRAG_AF118124_s_at	4170	MCL1	Myeloid cell leukemia sequence 1 (BCL2-related)
ADXCRAG_AF132197_s_at	26999	CYFIP2	Cytoplasmic FMR1 interacting protein 2
ADXCRAG_AF132197_x_at	26999	CYFIP2	Cytoplasmic FMR1 interacting protein 2
ADXCRAD_CD522843_s_at	64092	SAMSN1	SAM domain, SH3 domain and nuclear localization signals 1
ADXCRAG_AL050135_s_at	5993	RFX5	Regulatory factor X, 5 (influences HLA class II expression)
ADXCRAG_AL137572_s_at	116496	FAM129A	Family with sequence similarity 129, member A
ADXCRAG_AL390172_s_at	586	BCAT1	Branched chain aminotransferase 1, cytosolic
ADXCRAG_AL832674_s_at	81611	ANP32E	Acidic (leucine-rich) nuclear phosphoprotein 32 family, member E
ADXCRAG_AY057381_s_at	81611	ANP32E	Acidic (leucine-rich) nuclear phosphoprotein 32 family, member E
ADXCRAG_AY331789_at	NA	NA	
ADXCRAG_AY376241_s_at	7874	USP7	Ubiquitin specific peptidase 7 (herpes virus-associated)
ADXCRAG_AY426987_at	79864	C11orf63	Chromosome 11 open reading frame 63
ADXCRAG_AY572796_at	NA	NA	
ADXCRAG_BC001485_s_at	5880	RAC2	Ras-related C3 botulinum toxin substrate 2 (rho family, small GTP binding protein Rac2)
ADXCRAG_NM_033219_s_at	9830	TRIM14	Tripartite motif-containing 14
ADXCRAG_BC011762_s_at	26999	CYFIP2	Cytoplasmic FMR1 interacting protein 2
ADXCRAG_BC013120_at	5366	PMAIP1	Phorbol-12-myristate-13-acetate-induced protein 1
ADXCRAG_BC014267_s_at	10664	CTCF	CCCTC-binding factor (zinc finger protein)
ADXCRAD_CN367752_s_at	26502	NARF	Nuclear prelamin A recognition factor
ADXCRAG_BC018349_s_at	10320	IKZF1	IKAROS family zinc finger 1 (Ikaros)
ADXCRAG_BC025989_s_at	23395	LARS2	Leucyl-tRNA synthetase 2, mitochondrial
ADXCRAG_BC026073_s_at	57821	C1orf114	Chromosome 1 open reading frame 114
ADXCRAG_BC032349_s_at	23180	RFTN1	Raftlin, lipid raft linker 1
ADXCRAD_BM694731_s_at	9459	ARHGEF6	Rac/Cdc42 guanine nucleotide exchange factor (GEF) 6
ADXCRAG_BC039035_at	64084	CLSTN2	Calsyntenin 2
ADXCRAG_BC039035_x_at	64084	CLSTN2	Calsyntenin 2
ADXCRAG_BC050062_s_at	5791	PTPRE	Protein tyrosine phosphatase, receptor type, E
ADXCRAG_BC053878_at	7535	ZAP70	Zeta-chain (TCR) associated protein kinase 70kDa
ADXCRAG_BC053878_x_at	7535	ZAP70	Zeta-chain (TCR) associated protein kinase 70kDa
ADXCRAG_BX647660_s_at	9659	PDE4DIP	Phosphodiesterase 4D interacting protein (myomegalin)
ADXCRAG_NM_000418_s_at	3566	IL4R	Interleukin 4 receptor
ADXCRAG_NM_002832_s_at	5778	PTPN7	Protein tyrosine phosphatase, non-receptor type 7
ADXCRAD_BM795677_s_at	5788	PTPRC	Protein tyrosine phosphatase, receptor type, C
ADXCRAG_NM_006257_at	5588	PRKCQ	Protein kinase C, theta
ADXCRAD_AA234512_s_at	5588	PRKCQ	Protein kinase C, theta
ADXCRAG_NM_006504_at	5791	PTPRE	Protein tyrosine phosphatase, receptor type, E
ADXCRAD_BM725213_s_at	5791	PTPRE	Protein tyrosine phosphatase, receptor type, E
ADXCRAG_NM_012072_at	22918	CD93	CD93 molecule
ADXCRAG_NM_012072_s_at	22918	CD93	CD93 molecule
ADXCRAG_NM_015490_s_at	25956	SEC31B	SEC31 homolog B (S. cerevisiae)
ADXCRAG_NM_181359_at	3570	IL6R	Interleukin 6 receptor
ADXCRAG_U02297_s_at	6404	SELPLG	Selectin P ligand
ADXCRAG_U16296_at	7074	TIAM1	T-cell lymphoma invasion and metastasis 1
ADXCRAG_U17566_s_at	6573	SLC19A1	Solute carrier family 19 (folate transporter), member 1
ADXCRAG_U36500_s_at	11262	SP140	SP140 nuclear body protein
ADXCRAD_BG397984_s_at	11184	MAP4K1	Mitogen-activated protein kinase kinase kinase kinase 1
ADXCRAG_S57528_s_at	5696	PSMB8	Proteasome (prosome, macropain) subunit, beta type, 8 (large multifunctional peptidase 7)
ADXCRAG_XM_376764_s_at	10687	PNMA2	Paraneoplastic antigen MA2
ADXCRIH.2623.C1_s_at	4170	MCL1	Myeloid cell leukemia sequence 1 (BCL2-related)
ADXCRIH.625.C1_s_at	8531	CSDA	Cold shock domain protein A
ADXCRIH.3674.C1_at	5699	PSMB10	Proteasome (prosome, macropain) subunit, beta type, 10
ADXCRIH.1784.C1_s_at	5908	RAP1B	RAP1B, member of RAS oncogene family
ADXCRIH.2787.C1_s_at	10541	ANP32B	Acidic (leucine-rich) nuclear phosphoprotein 32 family, member B
ADXCRIH.3762.C1_at	9416	DDX23	DEAD (Asp-Glu-Ala-Asp) box polypeptide 23
ADXCRIH.2487.C1_s_at	51131	PHF11	PHD finger protein 11
ADXCRIH.455.C1_s_at	3726	JUNB	Jun B proto-oncogene
ADXCRIH.3101.C1_s_at	3428	IFI16	Interferon, gamma-inducible protein 16
ADXCRIH.1510.C1_s_at	3638	INSIG1	Insulin induced gene 1
ADXCRIH.138.C1_at	5771	PTPN2	Protein tyrosine phosphatase, non-receptor type 2
ADXCRIH.149.C1_s_at	3431	SP110	SP110 nuclear body protein
ADXCRIH.149.C1_x_at	3431	SP110	SP110 nuclear body protein
ADXCRIH.1587.C1_s_at	100533955	SENP3-EIF4A1	
ADXCRIH.2540.C1_at	5717	PSMD11	Proteasome (prosome, macropain) 26S subunit, non-ATPase, 11
ADXCRIH.3577.C1_at	4267	CD99	CD99 molecule
ADXCRIH.3577.C1_x_at	4267	CD99	CD99 molecule
ADXCRIH.1986.C1_s_at	391	RHOG	Ras homolog gene family, member G (rho G)
ADXCRIH.2963.C1_s_at	6774	STAT3	Signal transducer and activator of transcription 3 (acute-phase response factor)
ADXCRIH.3253.C1_s_at	5265	SERPINA1	Serpin peptidase inhibitor, clade A (alpha-1 antiproteinase, antitrypsin), member 1
ADXCRIH.2463.C2_at	4170	MCL1	Myeloid cell leukemia sequence 1 (BCL2-related)
ADXCRIH.2463.C2_s_at	4170	MCL1	Myeloid cell leukemia sequence 1 (BCL2-related)
ADXCRAD_BP342358_s_at	10209	EIF1	Eukaryotic translation initiation factor 1
ADXCRIH.116.C1_at	865	CBFB	Core-binding factor, beta subunit

ADXCRIH.116.C1_x_at	865	CBFB	Core-binding factor, beta subunit
ADXCRIH.69.C1_at	10376	TUBA1B	Tubulin, alpha 1b
ADXCRIH.69.C1_x_at	10376	TUBA1B	Tubulin, alpha 1b
ADXCRIH.187.C2_s_at	4673	NAP1L1	Nucleosome assembly protein 1-like 1
ADXCRIH.1214.C1_s_at	10236	HNRNPR	Heterogeneous nuclear ribonucleoprotein R
ADXCRIH.1078.C2_at	22987	SV2C	Synaptic vesicle glycoprotein 2C
ADXCRIH.1078.C2_x_at	22987	SV2C	Synaptic vesicle glycoprotein 2C
ADXCRIHRC.3674.C1_s_at	5699	PSMB10	Proteasome (prosome, macropain) subunit, beta type, 10
ADXCRIHRC.2540.C1_at	5717	PSMD11	Proteasome (prosome, macropain) 26S subunit, non-ATPase, 11
ADXCRIHRC.2540.C1_x_at	5717	PSMD11	Proteasome (prosome, macropain) 26S subunit, non-ATPase, 11
ADXCRAD_AL557216_s_at	865	CBFB	Core-binding factor, beta subunit
ADXCRIHRC.1078.C2_s_at	5908	RAP1B	RAP1B, member of RAS oncogene family
RDCR115_B08_at	64092	SAMSN1	SAM domain, SH3 domain and nuclear localization signals 1
RDCR115_B08_s_at	64092	SAMSN1	SAM domain, SH3 domain and nuclear localization signals 1
RDCR221_B10_at	23180	RFTN1	Raftlin, lipid raft linker 1
RDCR162_A06_s_at	23333	DPY19L1	Dpy-19-like 1 (C. elegans)
ADXCRPD.7962.C1_s_at	6573	SLC19A1	Solute carrier family 19 (folate transporter), member 1
ADXCRPD.1317.C1_at	10346	TRIM22	Tripartite motif-containing 22
ADXCRPD.1317.C1_x_at	10346	TRIM22	Tripartite motif-containing 22
ADXCRPD.10380.C1_at	7468	WHSC1	Wolf-Hirschhorn syndrome candidate 1
ADXCRPD.9220.C1_at	23082	PPRC1	Peroxisome proliferator-activated receptor gamma, coactivator-related 1
ADXCRPD.9220.C1_x_at	23082	PPRC1	Peroxisome proliferator-activated receptor gamma, coactivator-related 1
ADXCRPD.9260.C1_s_at	2767	GNA11	Guanine nucleotide binding protein (G protein), alpha 11 (Gq class)
ADXCRPD.9260.C1_x_at	2767	GNA11	Guanine nucleotide binding protein (G protein), alpha 11 (Gq class)
ADXCRPD.8623.C1_s_at	2769	GNA15	Guanine nucleotide binding protein (G protein), alpha 15 (Gq class)
ADXCRPD.1426.C1_at	NA	NA	
ADXCRPD.1437.C1_at	9535	GMFG	Glia maturation factor, gamma
ADXCRPD.18308.C1_at	9920	KBTBD11	Kelch repeat and BTB (POZ) domain containing 11
ADXCRPD.17780.C1_at	25956	SEC31B	SEC31 homolog B (S. cerevisiae)
ADXCRPD.2125.C1_at	64092	SAMSN1	SAM domain, SH3 domain and nuclear localization signals 1
ADXCRAD_BM806453_s_at	3428	IFI16	Interferon, gamma-inducible protein 16
ADXCRPD.11190.C1_at	3914	LAMB3	Laminin, beta 3
ADXCRPD.1564.C1_s_at	9416	DDX23	DEAD (Asp-Glu-Ala-Asp) box polypeptide 23
ADXCRPD.2201.C1_s_at	3431	SP110	SP110 nuclear body protein
ADXCRPD.10643.C1_at	7371	UCK2	Uridine-cytidine kinase 2
ADXCRPD.2309.C1_at	10346	TRIM22	Tripartite motif-containing 22
ADXCRPD.11382.C1_at	7874	USP7	Ubiquitin specific peptidase 7 (herpes virus-associated)
ADXCRPD.1749.C1_at	NA	NA	
ADXCRPD.3079.C1_s_at	4673	NAP1L1	Nucleosome assembly protein 1-like 1
ADXCRPD.3083.C1_s_at	116496	FAM129A	Family with sequence similarity 129, member A
ADXCRPD.1819.C1_at	963	CD53	CD53 molecule
ADXCRPD.9769.C1_at	55843	ARHGAP15	Rho GTPase activating protein 15
ADXCRPD.12103.C1_s_at	5771	PTPN2	Protein tyrosine phosphatase, non-receptor type 2
ADXCRPD.2533.C1_at	5771	PTPN2	Protein tyrosine phosphatase, non-receptor type 2
ADXCRPD.9810.C1_s_at	26999	CYFIP2	Cytoplasmic FMR1 interacting protein 2
ADXCRPD.9879.C1_s_at	7371	UCK2	Uridine-cytidine kinase 2
ADXCRPD.12254.C1_at	10664	CTCF	CCCTC-binding factor (zinc finger protein)
ADXCRPD.2751.C1_at	2335	FN1	Fibronectin 1
ADXCRPD.2751.C1_s_at	330	BIRC3	Baculoviral IAP repeat-containing 3
ADXCRPD.569.C1_s_at	5325	PLAGL1	Pleiomorphic adenoma gene-like 1
ADXCRPD.13006.C1_s_at	6774	STAT3	Signal transducer and activator of transcription 3 (acute-phase response factor)
ADXCRPD.4071.C1_at	9123	SLC16A3	Solute carrier family 16, member 3 (monocarboxylic acid transporter 4)
ADXCRPD.3499.C1_at	7040	TGFB1	Transforming growth factor, beta 1
ADXCRPD.734.C1_at	11262	SP140	SP140 nuclear body protein
ADXCRPD.734.C1_x_at	11262	SP140	SP140 nuclear body protein
ADXCRPD.793.C1_at	330	BIRC3	Baculoviral IAP repeat-containing 3
ADXCRPD.793.C1_x_at	330	BIRC3	Baculoviral IAP repeat-containing 3
ADXCRPD.891.C1_at	5698	PSMB9	Proteasome (prosome, macropain) subunit, beta type, 9 (large multifunctional peptidase 2)
ADXCRPD.3701.C1_at	963	CD53	CD53 molecule
ADXCRPD.12778.C1_at	5788	PTPRC	Protein tyrosine phosphatase, receptor type, C
ADXCRPD.936.C1_at	5366	PMAIP1	Phorbol-12-myristate-13-acetate-induced protein 1
ADXCRPD.14073.C1_at	286046	XKR6	XK, Kell blood group complex subunit-related family, member 6
ADXCRPD.14073.C1_s_at	57089	ENTPD7	
ADXCRPD.14073.C1_x_at	57089	ENTPD7	
ADXCRPD.5068.C1_at	5790	PTPRCAP	Protein tyrosine phosphatase, receptor type, C-associated protein
ADXCRPD.5068.C1_x_at	5790	PTPRCAP	Protein tyrosine phosphatase, receptor type, C-associated protein
ADXCRPD.5097.C1_s_at	7371	UCK2	Uridine-cytidine kinase 2
ADXCRPD.13500.C1_at	NA	NA	
ADXCRPD.13542.C1_at	3566	IL4R	Interleukin 4 receptor
ADXCRPD.13585.C1_at	6045	RNF2	Ring finger protein 2
ADXCRPD.12952.C1_at	5788	PTPRC	Protein tyrosine phosphatase, receptor type, C
ADXCRPD.5219.C1_at	NA	NA	
ADXCRPD.5219.C1_x_at	NA	NA	
ADXCRPD.5339.C1_s_at	5993	RFX5	Regulatory factor X, 5 (influences HLA class II expression)
ADXCRPD.15034.C1_s_at	9416	DDX23	DEAD (Asp-Glu-Ala-Asp) box polypeptide 23
ADXCRPD.14415.C1_s_at	7371	UCK2	Uridine-cytidine kinase 2
ADXCRPD.13867.C1_at	467	ATF3	Activating transcription factor 3
ADXCRPD.15116.C1_at	7371	UCK2	Uridine-cytidine kinase 2
ADXCRPD.6100.C1_at	10538	BATF	Basic leucine zipper transcription factor, ATF-like
ADXCRPD.14599.C1_at	7371	UCK2	Uridine-cytidine kinase 2
ADXCRPD.6256.C1_at	NA	NA	
ADXCRPD.5634.C1_at	NA	NA	
ADXCRPD.16013.C1_s_at	100533955	SENP3-EIF4A1	
ADXCRPD.14825.C1_at	80267	EDEM3	ER degradation enhancer, mannosidase alpha-like 3

ADXCRPD.6475.C1_s_at	3059	HCLS1	Hematopoietic cell-specific Lyn substrate 1
ADXCRPD.15558.C1_s_at	79726	WDR59	WD repeat domain 59
ADXCRPD.15731.C1_s_at	6774	STAT3	Signal transducer and activator of transcription 3 (acute-phase response factor)
ADXCRAD_BX107802_s_at	10460	TACC3	Transforming, acidic coiled-coil containing protein 3
ADXCRPD.17046.C1_at	23333	DPY19L1	Dpy-19-like 1 (C. elegans)
ADXCRPD.17046.C1_s_at	23333	DPY19L1	Dpy-19-like 1 (C. elegans)
ADXCRPD.16454.C1_at	7874	USP7	Ubiquitin specific peptidase 7 (herpes virus-associated)
ADXCRPD.7472.C1_s_at	9015	TAF1A	TATA box binding protein (TBP)-associated factor, RNA polymerase I, A, 48kDa
ADXCRAD_BG925633_s_at	4783	NFIL3	Nuclear factor, interleukin 3 regulated
ADXCRPD.8133.C1_at	7038	TG	Thyroglobulin
ADXCRPD.8173.C1_s_at	22918	CD93	CD93 molecule
ADXCRPD.7602.C1_s_at	6774	STAT3	Signal transducer and activator of transcription 3 (acute-phase response factor)
ADXCRPD.16668.C1_at	23082	PPRC1	Peroxisome proliferator-activated receptor gamma, coactivator-related 1
ADXCRPD.7685.C1_s_at	4170	MCL1	Myeloid cell leukemia sequence 1 (BCL2-related)
ADXCRPD.10135.C1_at	6624	FSCN1	Fascin homolog 1, actin-bundling protein (Strongylocentrotus purpuratus)
ADXCRPD.10135.C1_x_at	6624	FSCN1	Fascin homolog 1, actin-bundling protein (Strongylocentrotus purpuratus)
ADXCRAD_AL548133_s_at	6503	SLA	Src-like-adaptor
ADXCRPD.17460.C1_x_at	4242	MFNG	MFNG O-fucosylpeptide 3-beta-N-acetylglucosaminyltransferase
ADXCRPD.1208.C1_at	586	BCAT1	Branched chain aminotransferase 1, cytosolic
ADXCRPD.10268.C1_s_at	23333	DPY19L1	Dpy-19-like 1 (C. elegans)
ADXCRPD.1294.C1_s_at	467	ATF3	Activating transcription factor 3
ADXCRPD.250.C1_at	NA	NA	
ADXCRPD.2849.C2_at	10236	HNRNPR	Heterogeneous nuclear ribonucleoprotein R
ADXCRPD.139.C1_at	NA	NA	
ADXCRPD.367.C1_s_at	4673	NAP1L1	Nucleosome assembly protein 1-like 1
ADXCRPD.949.C1_s_at	7874	USP7	Ubiquitin specific peptidase 7 (herpes virus-associated)
ADXCRPDRC.10380.C1_s_at	7468	WHSC1	Wolf-Hirschhorn syndrome candidate 1
ADXCRPDRC.18308.C1_at	NA	NA	
ADXCRPDRC.17780.C1_at	160365	CLECL1	C-type lectin-like 1
ADXCRPDRC.17780.C1_s_at	25956	SEC31B	SEC31 homolog B (S. cerevisiae)
ADXCRPDRC.11382.C1_at	7874	USP7	Ubiquitin specific peptidase 7 (herpes virus-associated)
ADXCRPDRC.2453.C1_at	51076	CUTC	CutC copper transporter homolog (E. coli)
ADXCRPDRC.2453.C1_s_at	51076	CUTC	CutC copper transporter homolog (E. coli)
ADXCRPDRC.9769.C1_s_at	55843	ARHGAP15	Rho GTPase activating protein 15
ADXCRPDRC.12254.C1_s_at	10664	CTCF	CCCTC-binding factor (zinc finger protein)
ADXCRPDRC.4071.C1_at	NA	NA	
ADXCRAD_BU782064_s_at	7040	TGFB1	Transforming growth factor, beta 1
ADXCRPDRC.936.C1_s_at	5366	PMAIP1	Phorbol-12-myristate-13-acetate-induced protein 1
ADXCRPDRC.5097.C1_at	7371	UCK2	Uridine-cytidine kinase 2
ADXCRPDRC.5097.C1_x_at	7371	UCK2	Uridine-cytidine kinase 2
ADXCRPDRC.12952.C1_at	NA	NA	
ADXCRPDRC.12952.C1_s_at	5788	PTPRC	Protein tyrosine phosphatase, receptor type, C
ADXCRPDRC.13867.C1_s_at	467	ATF3	Activating transcription factor 3
ADXCRPDRC.16454.C1_at	663	BNIP2	BCL2/adenovirus E1B 19kDa interacting protein 2
ADXCRPDRC.16454.C1_s_at	7874	USP7	Ubiquitin specific peptidase 7 (herpes virus-associated)
ADXCRPDRC.8133.C1_at	6503	SLA	Src-like-adaptor
ADXCRAD_BX436824_s_at	6503	SLA	Src-like-adaptor
ADXCRPDRC.16668.C1_at	23082	PPRC1	Peroxisome proliferator-activated receptor gamma, coactivator-related 1
ADXCRPDRC.7895.C1_s_at	4242	MFNG	MFNG O-fucosylpeptide 3-beta-N-acetylglucosaminyltransferase
ADXCRPDRC.2849.C2_at	10236	HNRNPR	Heterogeneous nuclear ribonucleoprotein R
ADXCRPDRC.2569.C2_at	9830	TRIM14	Tripartite motif-containing 14
ADXCRSS.Hs#S1223446_at	2767	GNA11	Guanine nucleotide binding protein (G protein), alpha 11 (Gq class)
ADXCRSS.Hs#S5972058_at	NA	NA	
ADXCRSS.Hs#S2524484_at	26999	CYFIP2	Cytoplasmic FMR1 interacting protein 2
ADXCRSS.Hs#S2731818_at	54476	RNF216	Ring finger protein 216
ADXCRSS.Hs#S1919678_at	8531	CSDA	Cold shock domain protein A
ADXCRSS.Hs#S1751591_s_at	6774	STAT3	Signal transducer and activator of transcription 3 (acute-phase response factor)
ADXCRSS.Hs#S523708_at	3566	IL4R	Interleukin 4 receptor
ADXCRSS.Hs#S2990382_at	7874	USP7	Ubiquitin specific peptidase 7 (herpes virus-associated)
ADXCRSS.Hs#S2980215_s_at	6404	SELPLG	Selectin P ligand
ADXCRSS.Hs#S2978401_at	3607	FOXK2	Forkhead box K2
ADXCRSS.Hs#S3733207_at	26999	CYFIP2	Cytoplasmic FMR1 interacting protein 2
ADXCRSS.Hs#S2986318_at	23395	LARS2	Leucyl-tRNA synthetase 2, mitochondrial
ADXCRSS.Hs#S3742921_at	3566	IL4R	Interleukin 4 receptor
ADXCRIH.1957.C1_at	5696	PSMB8	Proteasome (prosome, macropain) subunit, beta type, 8 (large multifunctional peptidase 7)
ADXCRIH.1957.C2_at	5696	PSMB8	Proteasome (prosome, macropain) subunit, beta type, 8 (large multifunctional peptidase 7)
ADXCRIH.1957.C2_s_at	5696	PSMB8	Proteasome (prosome, macropain) subunit, beta type, 8 (large multifunctional peptidase 7)
ADXCRPD.1023.C1_at	3428	IFI16	Interferon, gamma-inducible protein 16
ADXCRPD.1900.C1_at	5724	PTAFR	
ADXCRPD.1900.C1_x_at	330	BIRC3	Baculoviral IAP repeat-containing 3
ADXCRPD.1900.C2_s_at	330	BIRC3	Baculoviral IAP repeat-containing 3
ADXCRPD.2569.C1_s_at	9830	TRIM14	Tripartite motif-containing 14
ADXCRPD.4854.C2_s_at	6890	TAP1	Transporter 1, ATP-binding cassette, sub-family B (MDR/TAP)
ADXCRAD_BQ654088_at	2899	GRIK3	Glutamate receptor, ionotropic, kainate 3
ADXCRAD_BQ654088_x_at	5551	PRF1	Perforin 1 (pore forming protein)
ADXCRAD_AW950959_at	11143	KAT7	
ADXCRAD_AW950959_s_at	7535	ZAP70	Zeta-chain (TCR) associated protein kinase 70kDa
ADXCRAD_BU198472_s_at	64092	SAMSN1	SAM domain, SH3 domain and nuclear localization signals 1
ADXCRAD_BP365482_s_at	4170	MCL1	Myeloid cell leukemia sequence 1 (BCL2-related)
ADXCRAD_BQ962674_at	4170	MCL1	Myeloid cell leukemia sequence 1 (BCL2-related)
ADXCRAD_BM712589_s_at	7468	WHSC1	Wolf-Hirschhorn syndrome candidate 1
ADXCRAD_BM755805_s_at	7468	WHSC1	Wolf-Hirschhorn syndrome candidate 1
ADXCRAD_BU956106_at	NA	NA	
ADXCRAD_BU191559_at	3638	INSIG1	Insulin induced gene 1

ADXCRAD_BU570382_at	3638	INSIG1	Insulin induced gene 1
ADXCRAD_BU570382_x_at	3638	INSIG1	Insulin induced gene 1
ADXCRAD_CD511474_at	27010	TPK1	Thiamin pyrophosphokinase 1
ADXCRAD_CD511474_s_at	27010	TPK1	Thiamin pyrophosphokinase 1
ADXCRAD_CD511474_x_at	27010	TPK1	Thiamin pyrophosphokinase 1
ADXCRAD_BG680099_at	NA	NA	
ADXCRAD_CB164277_at	10664	CTCF	CCCTC-binding factor (zinc finger protein)
ADXCRAD_BI256444_at	55260	TMEM143	Transmembrane protein 143
ADXCRAD_BI256444_x_at	55260	TMEM143	Transmembrane protein 143
ADXCRAD_BX389413_at	NA	NA	
ADXCRAD_BU953528_at	586	BCAT1	Branched chain aminotransferase 1, cytosolic
ADXCRAD_BU953528_x_at	586	BCAT1	Branched chain aminotransferase 1, cytosolic
ADXCRAD_BG393815_at	NA	NA	
ADXCRAD_BM919440_at	NA	NA	
ADXCRAD_BM919440_s_at	399	RHOH	Ras homolog gene family, member H
ADXCRAD_BG685498_at	10320	IKZF1	IKAROS family zinc finger 1 (Ikaros)
ADXCRAD_BG685498_x_at	10320	IKZF1	IKAROS family zinc finger 1 (Ikaros)
ADXCRAD_BQ082118_x_at	7024	TFCP2	Transcription factor CP2
ADXCRAD_BP313907_s_at	3570	IL6R	Interleukin 6 receptor
ADXCRAD_CN293583_at	9015	TAF1A	TATA box binding protein (TBP)-associated factor, RNA polymerase I, A, 48kDa
ADXCRAD_AL561208_at	865	CBFB	Core-binding factor, beta subunit
ADXCRAD_AL561208_x_at	865	CBFB	Core-binding factor, beta subunit
ADXCRAD_CV027630_s_at	5880	RAC2	Ras-related C3 botulinum toxin substrate 2 (rho family, small GTP binding protein Rac2)
ADXCRAD_BP220549_at	4673	NAP1L1	Nucleosome assembly protein 1-like 1
ADXCRAD_BP220549_x_at	4673	NAP1L1	Nucleosome assembly protein 1-like 1
ADXCRAD_CX788441_at	10236	HNRNPR	Heterogeneous nuclear ribonucleoprotein R
ADXCRAD_CX788441_x_at	10236	HNRNPR	Heterogeneous nuclear ribonucleoprotein R
ADXCRAD_CD252021_x_at	3428	IFI16	Interferon, gamma-inducible protein 16
ADXCRAD_CN390223_s_at	7468	WHSC1	Wolf-Hirschhorn syndrome candidate 1
ADXCRAD_CN390208_s_at	7468	WHSC1	Wolf-Hirschhorn syndrome candidate 1
ADXCRAD_BF970701_at	NA	NA	
ADXCRAD_CA439064_at	7874	USP7	Ubiquitin specific peptidase 7 (herpes virus-associated)
ADXCRAD_CA942280_s_at	5325	PLAGL1	Pleiomorphic adenoma gene-like 1
ADXCRAD_CK002790_at	100288142	LOC100288142	
ADXCRAD_CK002790_x_at	100288142	LOC100288142	
ADXCRAD_BI818193_s_at	6404	SELPLG	Selectin P ligand
ADXCRAD_CX761856_s_at	10209	EIF1	Eukaryotic translation initiation factor 1
ADXCRAD_BX436427_s_at	5788	PTPRC	Protein tyrosine phosphatase, receptor type, C
ADXCRAD_BU607292_at	23333	DPY19L1	Dpy-19-like 1 (C. elegans)
ADXCRAD_BU607292_x_at	23333	DPY19L1	Dpy-19-like 1 (C. elegans)
ADXCRAD_CN296684_s_at	3178	HNRNPA1	Heterogeneous nuclear ribonucleoprotein A1
ADXCRAD_BX457462_s_at	915	CD3D	CD3d molecule, delta (CD3-TCR complex)
ADXCRAD_CX870261_s_at	4673	NAP1L1	Nucleosome assembly protein 1-like 1
ADXCRAD_CX866513_at	4170	MCL1	Myeloid cell leukemia sequence 1 (BCL2-related)
ADXCRAD_BU522109_at	100128124	HGC6.3	
ADXCRAD_BG570659_at	53346	TM6SF1	Transmembrane 6 superfamily member 1
ADXCRAD_AV725374_at	26999	CYFIP2	Cytoplasmic FMR1 interacting protein 2
ADXCRAD_CR745173_s_at	27010	TPK1	Thiamin pyrophosphokinase 1
ADXCRAD_BE621153_at	NA	NA	
ADXCRAD_AA393120_at	79726	WDR59	WD repeat domain 59
ADXCRAD_CA437914_at	7874	USP7	Ubiquitin specific peptidase 7 (herpes virus-associated)
ADXCRAD_AL541939_s_at	3638	INSIG1	Insulin induced gene 1
ADXCRAD_BG192476_s_at	100288142	LOC100288142	
ADXCRAD_BG756941_at	100507377	LOC100507377	
ADXCRAD_BG546180_s_at	5788	PTPRC	Protein tyrosine phosphatase, receptor type, C
ADXCRAD_BQ884238_at	4673	NAP1L1	Nucleosome assembly protein 1-like 1
ADXCRAD_BX409785_x_at	25897	RNF19A	Ring finger protein 19A
ADXCRAD_BU073065_s_at	10687	PNMA2	Paraneoplastic antigen MA2
ADXCRAD_BM808025_at	7074	TIAM1	T-cell lymphoma invasion and metastasis 1
ADXCRAD_BF981280_at	NA	NA	
ADXCRAD_BM472661_at	586	BCAT1	Branched chain aminotransferase 1, cytosolic
ADXCRAD_BM472661_x_at	586	BCAT1	Branched chain aminotransferase 1, cytosolic
ADXCRAD_CN391960_at	81611	ANP32E	Acidic (leucine-rich) nuclear phosphoprotein 32 family, member E
ADXCRAD_AJ708655_s_at	6624	FSCN1	Fascin homolog 1, actin-bundling protein (Strongylocentrotus purpuratus)
ADXCRAD_BG388017_x_at	5778	PTPN7	Protein tyrosine phosphatase, non-receptor type 7
ADXCRAD_BG757090_at	NA	NA	
ADXCRAD_BG757090_x_at	NA	NA	
ADXCRAD_BM830891_at	55013	CCDC109B	Coiled-coil domain containing 109B
ADXCRAD_BE278749_s_at	100533184	ARHGAP19-SLIT1	
ADXCRAD_BI222503_at	9830	TRIM14	Tripartite motif-containing 14
ADXCRAD_BU150433_at	NA	NA	
ADXCRAD_BU150433_x_at	22918	CD93	CD93 molecule
ADXCRAD_BG285017_at	53346	TM6SF1	Transmembrane 6 superfamily member 1
ADXCRAD_BM456472_s_at	10320	IKZF1	IKAROS family zinc finger 1 (Ikaros)
ADXCRAD_NM_080424_s_at	3431	SP110	SP110 nuclear body protein
ADXCRAD_NM_005546_at	3702	ITK	IL2-inducible T-cell kinase
ADXCRAD_NM_004843_at	56776	FMN2	Formin 2
ADXCRAD_NM_014867_at	9920	KBTBD11	Kelch repeat and BTB (POZ) domain containing 11
ADXCRAD_NM_198053_at	919	CD247	CD247 molecule
ADXCRAD_NM_018015_s_at	55086	CXorf57	Chromosome X open reading frame 57
ADXCRAD_NM_002405_at	4242	MFNG	MFNG O-fucosylpeptide 3-beta-N-acetylglucosaminyltransferase
ADXCRAD_NM_004946_at	1794	DOCK2	Dedicator of cytokinesis 2
ADXCRAD_AB078026_s_at	467	ATF3	Activating transcription factor 3
ADXCRAD_AK095268_x_at	8729	GBF1	Golgi-specific brefeldin A resistance factor 1

ADXCRAD_BC031525_at	5788	PTPRC	Protein tyrosine phosphatase, receptor type, C
ADXCRAD_NM_000660_at	3183	HNRNPC	Heterogeneous nuclear ribonucleoprotein C (C1/C2)
ADXCRAD_NM_000660_x_at	3183	HNRNPC	Heterogeneous nuclear ribonucleoprotein C (C1/C2)
ADXCRAD_AI697657_s_at	81611	ANP32E	Acidic (leucine-rich) nuclear phosphoprotein 32 family, member E
ADXCRAD_AI972599_at	7874	USP7	Ubiquitin specific peptidase 7 (herpes virus-associated)
ADXCRAD_U37546_x_at	330	BIRC3	Baculoviral IAP repeat-containing 3
ADXCRAD_AK025190_x_at	57590	WDFY1	WD repeat and FYVE domain containing 1
ADXCRAD_BF724558_at	574406	C1orf138	Chromosome 1 open reading frame 138
ADXCRAD_BF724558_x_at	574406	C1orf138	Chromosome 1 open reading frame 138
ADXCRAD_AW293276_at	963	CD53	CD53 molecule
ADXCRAD_AI510829_at	55843	ARHGAP15	Rho GTPase activating protein 15
ADXCRAD_AA805622_at	330	BIRC3	Baculoviral IAP repeat-containing 3
ADXCRAD_BF447983_at	23333	DPY19L1	Dpy-19-like 1 (C. elegans)
ADXCRAD_AI682905_x_at	5573	PRKAR1A	Protein kinase, cAMP-dependent, regulatory, type I, alpha (tissue specific extinguisher 1)
ADXCRAD_BC004908_at	6624	FSCN1	Fascin homolog 1, actin-bundling protein (Strongylocentrotus purpuratus)
ADXCRAD_BC004908_s_at	6624	FSCN1	Fascin homolog 1, actin-bundling protein (Strongylocentrotus purpuratus)
ADXCRAD_AI445650_at	5551	PRF1	Perforin 1 (pore forming protein)
ADXCRAD_H24473_at	100286793	LOC100286793	
ADXCRAD_H24473_s_at	100286793	LOC100286793	
ADXCRAD_AA767714_at	3566	IL4R	Interleukin 4 receptor

S1 Table part B

probeset_id	entrez_id	hgnc_symbol	Description
ADXCRAG_AF006751_s_at	6238	RRBP1	Ribosome binding protein 1 homolog 180kDa (dog)
ADXCRAG_AF077226_at	8895	CPNE3	Copine III
ADXCRAG_AF098865_at	6713	SQLE	Squalene epoxidase
ADXCRAD_CB106889_s_at	6713	SQLE	Squalene epoxidase
ADXCRAG_AF233882_x_at	3728	JUP	Junction plakoglobin
ADXCRAG_AF480883_at	9499	MYOT	Myotilin
ADXCRAG_AL833398_s_at	1488	CTBP2	C-terminal binding protein 2
ADXCRAG_BC002522_s_at	51111	SUV420H1	Suppressor of variegation 4-20 homolog 1 (Drosophila)
ADXCRAG_BC005274_at	8821	INPP4B	Inositol polyphosphate-4-phosphatase, type II, 105kDa
ADXCRAG_BC009700_x_at	6238	RRBP1	Ribosome binding protein 1 homolog 180kDa (dog)
ADXCRAG_BC011987_s_at	87	ACTN1	Actinin, alpha 1
ADXCRAG_BC032656_s_at	6934	TCF7L2	Transcription factor 7-like 2 (T-cell specific, HMG-box)
ADXCRAG_BC045655_s_at	27346	TMEM97	Transmembrane protein 97
ADXCRAD_CB217549_s_at	7026	NR2F2	Nuclear receptor subfamily 2, group F, member 2
ADXCRAG_L14837_at	7082	TJP1	Tight junction protein 1 (zona occludens 1)
ADXCRAD_BM352913_s_at	7082	TJP1	Tight junction protein 1 (zona occludens 1)
ADXCRAG_NM_003243_s_at	7049	TGFBR3	Transforming growth factor, beta receptor III
ADXCRAD_CD678016_s_at	8613	PPAP2B	Phosphatidic acid phosphatase type 2B
ADXCRAG_NM_005558_at	3898	LAD1	Ladinin 1
ADXCRAD_BI837070_at	NA	NA	
ADXCRAD_BI837070_s_at	51599	LSR	Lipolysis stimulated lipoprotein receptor
ADXCRAG_U60975_s_at	6653	SORL1	Sortilin-related receptor, L(DLR class) A repeats-containing
ADXCRAG_U70063_at	427	ASAH1	N-acylsphingosine amidohydrolase (acid ceramidase) 1
ADXCRAG_U70063_s_at	427	ASAH1	N-acylsphingosine amidohydrolase (acid ceramidase) 1
ADXCRIH.3628.C1_s_at	3898	LAD1	Ladinin 1
ADXCRIH.2736.C1_s_at	9516	LITAF	Lipopolysaccharide-induced TNF factor
ADXCRIH.1401.C1_s_at	27328	PCDH11X	Protocadherin 11 X-linked
ADXCRIH.2082.C1_s_at	3728	JUP	Junction plakoglobin
ADXCRIH.1454.C1_s_at	55353	LAPTM4B	Lysosomal associated protein transmembrane 4 beta
ADXCRIH.878.C1_s_at	5269	SERPINB6	Serpin peptidase inhibitor, clade B (ovalbumin), member 6
ADXCRIH.2137.C1_at	1829	DSG2	Desmoglein 2
ADXCRIH.3146.C1_at	126353	C19orf21	Chromosome 19 open reading frame 21
ADXCRIH.3170.C1_at	7049	TGFBR3	Transforming growth factor, beta receptor III
ADXCRIH.1931.C1_at	1488	CTBP2	C-terminal binding protein 2
ADXCRIH.1931.C1_s_at	1488	CTBP2	C-terminal binding protein 2
ADXCRIH.3560.C1_at	11015	KDELR3	
ADXCRIH.2935.C1_s_at	8895	CPNE3	Copine III
ADXCRIH.3943.C1_s_at	7082	TJP1	Tight junction protein 1 (zona occludens 1)
ADXCRIH.2240.C1_at	2879	GPX4	Glutathione peroxidase 4 (phospholipid hydroperoxidase)
ADXCRIH.2281.C1_at	2810	SFN	Stratifin
ADXCRIH.2281.C1_x_at	2810	SFN	Stratifin
ADXCRIH.184.C1_at	3958	LGALS3	Lectin, galactoside-binding, soluble, 3
ADXCRIH.184.C1_x_at	3958	LGALS3	Lectin, galactoside-binding, soluble, 3
ADXCRIHRC.184.C1_at	3958	LGALS3	Lectin, galactoside-binding, soluble, 3
ADXCRIHRC.184.C1_s_at	3958	LGALS3	Lectin, galactoside-binding, soluble, 3
RDCR260_H04_s_at	9516	LITAF	Lipopolysaccharide-induced TNF factor
RDCR087_E11_s_at	51111	SUV420H1	Suppressor of variegation 4-20 homolog 1 (Drosophila)
ADXCRPD.16952.C1_at	5269	SERPINB6	Serpin peptidase inhibitor, clade B (ovalbumin), member 6
ADXCRPD.11126.C1_at	11015	KDELR3	
ADXCRPD.11180.C1_at	5269	SERPINB6	Serpin peptidase inhibitor, clade B (ovalbumin), member 6
ADXCRPD.8849.C1_at	8821	INPP4B	Inositol polyphosphate-4-phosphatase, type II, 105kDa
ADXCRPD.10614.C1_at	79083	MLPH	Melanophilin
ADXCRPD.9737.C1_at	2810	SFN	Stratifin
ADXCRPD.11593.C1_at	7082	TJP1	Tight junction protein 1 (zona occludens 1)
ADXCRPD.1966.C1_at	6934	TCF7L2	Transcription factor 7-like 2 (T-cell specific, HMG-box)
ADXCRPD.553.C1_at	9516	LITAF	Lipopolysaccharide-induced TNF factor
ADXCRPD.3405.C1_at	4041	LRP5	Low density lipoprotein receptor-related protein 5
ADXCRPD.13135.C1_at	79083	MLPH	Melanophilin
ADXCRPD.13135.C1_x_at	79083	MLPH	Melanophilin

ADXCRPD.3501.C1_s_at	595	CCND1	Cyclin D1
ADXCRAD_BM551840_x_at	595	CCND1	Cyclin D1
ADXCRPD.3569.C1_at	11015	KDELR3	
ADXCRPD.12698.C1_at	8821	INPP4B	Inositol polyphosphate-4-phosphatase, type II, 105kDa
ADXCRPD.12698.C1_x_at	8821	INPP4B	Inositol polyphosphate-4-phosphatase, type II, 105kDa
ADXCRPD.13409.C1_at	1829	DSG2	Desmoglein 2
ADXCRPD.13412.C1_s_at	8613	PPAP2B	Phosphatidic acid phosphatase type 2B
ADXCRPD.13497.C1_at	1829	DSG2	Desmoglein 2
ADXCRPD.3980.C1_s_at	595	CCND1	Cyclin D1
ADXCRPD.4673.C1_at	7026	NR2F2	Nuclear receptor subfamily 2, group F, member 2
ADXCRPD.15019.C1_at	79083	MLPH	Melanophilin
ADXCRPD.13939.C1_at	595	CCND1	Cyclin D1
ADXCRPD.16054.C1_at	595	CCND1	Cyclin D1
ADXCRPD.14975.C1_s_at	7082	TJP1	Tight junction protein 1 (zona occludens 1)
ADXCRPD.8158.C1_s_at	51111	SUV420H1	Suppressor of variegation 4-20 homolog 1 (Drosophila)
ADXCRPD.15960.C1_at	595	CCND1	Cyclin D1
ADXCRPD.8241.C1_at	6713	SQLE	Squalene epoxidase
ADXCRPD.16751.C1_at	6934	TCF7L2	Transcription factor 7-like 2 (T-cell specific, HMG-box)
ADXCRPD.18092.C1_at	6653	SORL1	Sortilin-related receptor, L(DLR class) A repeats-containing
ADXCRPD.7803.C1_s_at	6238	RRBP1	Ribosome binding protein 1 homolog 180kDa (dog)
ADXCRPD.1115.C1_s_at	79083	MLPH	Melanophilin
ADXCRPDRC.11126.C1_at	11015	KDELR3	
ADXCRPDRC.9737.C1_at	2810	SFN	Stratifin
ADXCRPDRC.9737.C1_s_at	2810	SFN	Stratifin
ADXCRPDRC.9737.C1_x_at	2810	SFN	Stratifin
ADXCRPDRC.11593.C1_s_at	7082	TJP1	Tight junction protein 1 (zona occludens 1)
ADXCRPDRC.4673.C1_s_at	7026	NR2F2	Nuclear receptor subfamily 2, group F, member 2
ADXCRPDRC.16054.C1_at	595	CCND1	Cyclin D1
ADXCRPDRC.8241.C1_s_at	6713	SQLE	Squalene epoxidase
ADXCRSS.Hs#S1444631_at	3958	LGALS3	Lectin, galactoside-binding, soluble, 3
ADXCRSS.Hs#S11047314_at	6934	TCF7L2	Transcription factor 7-like 2 (T-cell specific, HMG-box)
ADXCRSS.Hs#S1748600_at	427	ASAH1	N-acylsphingosine amidohydrolase (acid ceramidase) 1
ADXCRSS.Hs#S1920919_at	6934	TCF7L2	Transcription factor 7-like 2 (T-cell specific, HMG-box)
ADXCRSS.Hs#S1921395_at	79083	MLPH	Melanophilin
ADXCRSS.Hs#S1910362_at	4041	LRP5	Low density lipoprotein receptor-related protein 5
ADXCRSS.Hs#S524340_at	8613	PPAP2B	Phosphatidic acid phosphatase type 2B
ADXCRSS.Hs#S2577958_at	51111	SUV420H1	Suppressor of variegation 4-20 homolog 1 (Drosophila)
ADXCRSS.Hs#S2577958_s_at	51111	SUV420H1	Suppressor of variegation 4-20 homolog 1 (Drosophila)
ADXCRSS.Hs#S3732256_at	10521	DDX17	DEAD (Asp-Glu-Ala-Asp) box polypeptide 17
ADXCRSS.Hs#S2985193_at	55291	PPP6R3	
ADXCRSS.Hs#S2984021_at	7082	TJP1	Tight junction protein 1 (zona occludens 1)
ADXCRSS.Hs#S2986702_at	5269	SERPINB6	Serpin peptidase inhibitor, clade B (ovalbumin), member 6
ADXCRSS.Hs#S3008063_at	1488	CTBP2	C-terminal binding protein 2
ADXCRSS.Hs#S3013361_at	6934	TCF7L2	Transcription factor 7-like 2 (T-cell specific, HMG-box)
ADXCRSS.Hs#S3011054_at	55291	PPP6R3	
ADXCRSS.Hs#S624533_s_at	7049	TGFBR3	Transforming growth factor, beta receptor III
ADXCRSS.Hs#S3742718_at	6934	TCF7L2	Transcription factor 7-like 2 (T-cell specific, HMG-box)
ADXCRSS.Hs#S3748650_at	8821	INPP4B	Inositol polyphosphate-4-phosphatase, type II, 105kDa
ADXCRSS.Hs#S3748482_at	87	ACTN1	Actinin, alpha 1
ADXCRSS.Hs#S3891559_at	NA	NA	
ADXCRAD_BQ887548_s_at	1829	DSG2	Desmoglein 2
ADXCRAD_BE171045_s_at	1829	DSG2	Desmoglein 2
ADXCRAD_BX410303_at	6934	TCF7L2	Transcription factor 7-like 2 (T-cell specific, HMG-box)
ADXCRAD_BX410303_x_at	9516	LITAF	Lipopolysaccharide-induced TNF factor
ADXCRAD_BQ722638_at	NA	NA	
ADXCRAD_CB159041_at	6238	RRBP1	Ribosome binding protein 1 homolog 180kDa (dog)
ADXCRAD_CB159041_s_at	6238	RRBP1	Ribosome binding protein 1 homolog 180kDa (dog)
ADXCRAD_BQ778389_at	6238	RRBP1	Ribosome binding protein 1 homolog 180kDa (dog)
ADXCRAD_BQ931525_at	NA	NA	
ADXCRAD_BQ931525_x_at	23245	ASTN2	Astrotactin 2
ADXCRAD_BG614034_at	1488	CTBP2	C-terminal binding protein 2
ADXCRAD_BG614034_x_at	1488	CTBP2	C-terminal binding protein 2
ADXCRAD_CB852249_at	8821	INPP4B	Inositol polyphosphate-4-phosphatase, type II, 105kDa
ADXCRAD_BP222406_at	8895	CPNE3	Copine III
ADXCRAD_BP222406_x_at	8895	CPNE3	Copine III
ADXCRAD_BF664880_at	NA	NA	
ADXCRAD_CX871590_s_at	87	ACTN1	Actinin, alpha 1
ADXCRAD_BX089897_s_at	6653	SORL1	Sortilin-related receptor, L(DLR class) A repeats-containing
ADXCRAD_CB956496_at	7026	NR2F2	Nuclear receptor subfamily 2, group F, member 2
ADXCRAD_CB956496_x_at	7026	NR2F2	Nuclear receptor subfamily 2, group F, member 2
ADXCRAD_BM790129_at	4041	LRP5	Low density lipoprotein receptor-related protein 5
ADXCRAD_CN295781_s_at	6576	SLC25A1	Solute carrier family 25 (mitochondrial carrier; citrate transporter), member 1
ADXCRAD_CX866865_s_at	1488	CTBP2	C-terminal binding protein 2
ADXCRAD_CX867137_s_at	27346	TMEM97	Transmembrane protein 97
ADXCRAD_AU118429_s_at	6934	TCF7L2	Transcription factor 7-like 2 (T-cell specific, HMG-box)
ADXCRAD_BU857068_at	NA	NA	
ADXCRAD_BU173366_s_at	6934	TCF7L2	Transcription factor 7-like 2 (T-cell specific, HMG-box)
ADXCRAD_BM674772_at	7082	TJP1	Tight junction protein 1 (zona occludens 1)
ADXCRAD_BM674772_s_at	7082	TJP1	Tight junction protein 1 (zona occludens 1)
ADXCRAD_CN262594_s_at	6238	RRBP1	Ribosome binding protein 1 homolog 180kDa (dog)
ADXCRAD_CB153171_s_at	8895	CPNE3	Copine III
ADXCRAD_BG199926_s_at	8821	INPP4B	Inositol polyphosphate-4-phosphatase, type II, 105kDa
ADXCRAD_BG199926_x_at	8821	INPP4B	Inositol polyphosphate-4-phosphatase, type II, 105kDa
ADXCRAD_BF677896_at	NA	NA	

ADXCRAD_BF677896_x_at	NA	NA	
ADXCRAD_BQ436873_at	595	CCND1	Cyclin D1
ADXCRAD_AV758440_at	6934	TCF7L2	Transcription factor 7-like 2 (T-cell specific, HMG-box)
ADXCRAD_CN372474_at	7082	TJP1	Tight junction protein 1 (zona occludens 1)
ADXCRAD_BQ186493_s_at	427	ASAH1	N-acylsphingosine amidohydrolase (acid ceramidase) 1
ADXCRAD_BM714793_at	87	ACTN1	Actinin, alpha 1
ADXCRAD_CN335041_at	2810	SFN	Stratifin
ADXCRAD_CN335041_s_at	2810	SFN	Stratifin
ADXCRAD_CN335041_x_at	2810	SFN	Stratifin
ADXCRAD_NM_017635_at	51111	SUV420H1	Suppressor of variegation 4-20 homolog 1 (Drosophila)
ADXCRAD_NM_017635_x_at	51111	SUV420H1	Suppressor of variegation 4-20 homolog 1 (Drosophila)
ADXCRAD_BU754109_at	6713	SQLE	Squalene epoxidase
ADXCRAD_M95178_at	5054	SERPINE1	Serpin peptidase inhibitor, clade E (nexin, plasminogen activator inhibitor type 1), member 1
ADXCRAD_M95178_x_at	5054	SERPINE1	Serpin peptidase inhibitor, clade E (nexin, plasminogen activator inhibitor type 1), member 1
ADXCRAD_BE646396_at	6238	RRBP1	Ribosome binding protein 1 homolog 180kDa (dog)
ADXCRAD_BE646396_s_at	6238	RRBP1	Ribosome binding protein 1 homolog 180kDa (dog)
ADXCRAD_AK024129_x_at	1488	CTBP2	C-terminal binding protein 2
ADXCRAD_AL109707_at	7082	TJP1	Tight junction protein 1 (zona occludens 1)
ADXCRAD_AW268884_at	7049	TGFBR3	Transforming growth factor, beta receptor III
ADXCRAD_BF979497_at	NA	NA	
ADXCRAD_AA664011_at	6934	TCF7L2	Transcription factor 7-like 2 (T-cell specific, HMG-box)
ADXCRAD_BF439431_x_at	8504	PEX3	Peroxisomal biogenesis factor 3
ADXCRAD_AW173166_at	7082	TJP1	Tight junction protein 1 (zona occludens 1)
