# Supplementary material for: Cell Line Derived 5-FU and Irinotecan Drug-Sensitivity Profiles Evaluated in Adjuvant Colon Cancer Trial Data
Source: PLoS One. 2016 May 12;11(5):e0155123. doi: 10.1371/journal.pone.0155123 (PMC4865183; doi:10.1371/journal.pone.0155123)
Supplement: S2 Table — Part A. Probe sets for genes overexpressed in cell lines sensitive to 5-FU. The table shows the Almac probe sets that corresponds to the genes identified in cell lines. Note that some genes have several matching probe sets, the mapping is neither unique nor inambiguous. Part B. Probe sets for genes overexpressed in cell lines resistant to 5-FU. The table shows the Almac probe sets that corresponds to the genes identified in cell lines. Note that some genes have several matching probe sets, the mapping is neither unique nor unambiguous. (PDF) [file pone.0155123.s004.pdf]

S2 Table part A

probeset_id	entrez_id	hgnc_symbol	Description
200060_s_at	10921	RNPS1	RNA binding protein S1, serine-rich domain
200064_at	3326	HSP90AB1	Heat shock protein 90kDa alpha (cytosolic), class B member 1
ADXCRAD_AL583494_s_at	51116	MRPS2	Mitochondrial ribosomal protein S2
ADXCRAD_BM824056_s_at	23590	PDSS1	Prenyl (decaprenyl) diphosphate synthase, subunit 1
ADXCRAG_AJ250915_at	3329	HSPD1	Heat shock 60kDa protein 1 (chaperonin)
ADXCRAG_AL110194_s_at	3146	HMGB1	High-mobility group box 1
ADXCRAG_AL832032_at	400084	LOC400084	Hypothetical gene supported by AK057632; AL137270; BC057846
ADXCRAG_AY338490_at	2936	GSR	Glutathione reductase
ADXCRAG_AY526322_at	25888	ZNF473	Zinc finger protein 473
ADXCRAD_CX873763_s_at	5226	PGD	Phosphogluconate dehydrogenase
ADXCRAD_CN389613_s_at	8607	RUVBL1	RuvB-like 1 (E. coli)
ADXCRAG_BC005917_at	2960	GTF2E1	General transcription factor IIE, polypeptide 1, alpha 56kDa
ADXCRAG_BC022487_s_at	2729	GCLC	Glutamate-cysteine ligase, catalytic subunit
ADXCRAD_CD013938_s_at	1644	DDC	Dopa decarboxylase (aromatic L-amino acid decarboxylase)
ADXCRAG_NM_001149_s_at	288	ANK3	Ankyrin 3, node of Ranvier (ankyrin G)
ADXCRAD_CR738919_s_at	2729	GCLC	Glutamate-cysteine ligase, catalytic subunit
ADXCRAG_NM_004104_s_at	2194	FASN	Fatty acid synthase
ADXCRAG_NM_014456_s_at	27250	PDCD4	Programmed cell death 4 (neoplastic transformation inhibitor)
ADXCRAG_NM_033429_s_at	91860	CALML4	Calmodulin-like 4
ADXCRAD_CN272683_s_at	7112	TMPO	Thymopoietin
ADXCRAG_U17566_s_at	6573	SLC19A1	Solute carrier family 19 (folate transporter), member 1
ADXCRAG_U51677_x_at	3146	HMGB1	High-mobility group box 1
ADXCRAG_U88837_s_at	10985	GCN1L1	GCN1 general control of amino-acid synthesis 1-like 1 (yeast)
ADXCRAG_XM_0457920_at	10985	GCN1L1	GCN1 general control of amino-acid synthesis 1-like 1 (yeast)
ADXCRAD_BU599568_s_at	10985	GCN1L1	GCN1 general control of amino-acid synthesis 1-like 1 (yeast)
ADXCRIH.2614.C1_s_at	10247	HRSP12	Heat-responsive protein 12
ADXCRIH.3279.C1_x_at	3326	HSP90AB1	Heat shock protein 90kDa alpha (cytosolic), class B member 1
ADXCRIH.2640.C1_s_at	4609	MYC	V-myc myelocytomatosis viral oncogene homolog (avian)
ADXCRIH.651.C1_s_at	2114	ETS2	V-ets erythroblastosis virus E26 oncogene homolog 2 (avian)
ADXCRIH.1394.C1_s_at	1535	CYBA	Cytochrome b-245, alpha polypeptide
ADXCRIH.2353.C1_at	1992	SERPINB1	Serpin peptidase inhibitor, clade B (ovalbumin), member 1
ADXCRIH.2353.C1_s_at	1992	SERPINB1	Serpin peptidase inhibitor, clade B (ovalbumin), member 1
ADXCRIH.324.C1_s_at	7295	TXN	Thioredoxin
ADXCRIH.1715.C1_at	7203	CCT3	Chaperonin containing TCP1, subunit 3 (gamma)
ADXCRIH.333.C1_s_at	56667	MUC13	Mucin 13, cell surface associated
ADXCRIH.3349.C1_at	23019	CNOT1	CCR4-NOT transcription complex, subunit 1
ADXCRIH.2766.C1_at	23019	CNOT1	CCR4-NOT transcription complex, subunit 1
ADXCRIH.2766.C1_s_at	23019	CNOT1	CCR4-NOT transcription complex, subunit 1
ADXCRIH.2766.C1_x_at	23019	CNOT1	CCR4-NOT transcription complex, subunit 1
ADXCRIH.2777.C1_s_at	57402	S100A14	S100 calcium binding protein A14
ADXCRIH.2787.C1_s_at	10541	ANP32B	Acidic (leucine-rich) nuclear phosphoprotein 32 family, member B
ADXCRIH.2001.C1_s_at	54948	MRPL16	Mitochondrial ribosomal protein L16
ADXCRIH.1094.C1_s_at	7284	TUFM	Tu translation elongation factor, mitochondrial
ADXCRIH.789.C1_s_at	2877	GPX2	Glutathione peroxidase 2 (gastrointestinal)
ADXCRIH.3783.C1_s_at	2936	GSR	Glutathione reductase
ADXCRIH.1462.C1_at	60488	MRPS35	Mitochondrial ribosomal protein S35
ADXCRIH.445.CB1_s_at	6122	RPL3	Ribosomal protein L3
ADXCRIH.469.C1_at	29880	ALG5	Asparagine-linked glycosylation 5 homolog (S. cerevisiae, dolichyl-phosphate beta-glucosyltransferase)
ADXCRAD_NM_017627_s_at	7178	TPT1	Tumor protein, translationally-controlled 1
ADXCRIH.18.CB1_x_at	100885848	PTGES3L	
ADXCRIH.2825.C1_s_at	9368	SLC9A3R1	Solute carrier family 9 (sodium/hydrogen exchanger), member 3 regulator 1
ADXCRIH.498.C1_s_at	80142	PTGES2	Prostaglandin E synthase 2
ADXCRIH.1894.C1_s_at	79023	NUP37	Nucleoporin 37kDa
ADXCRIH.1103.C1_s_at	4706	NDUFAB1	NADH dehydrogenase (ubiquinone) 1, alpha/beta subcomplex, 1, 8kDa
ADXCRIH.1160.C1_s_at	9245	GCNT3	Glucosaminyl (N-acetyl) transferase 3, mucin type
ADXCRIH.3851.C1_at	30968	STOML2	Stomatin (EPB72)-like 2
ADXCRIH.873.C1_s_at	10797	MTHFD2	Methylenetetrahydrofolate dehydrogenase (NADP+ dependent) 2, methenyltetrahydrofolate cyclohydrolase
ADXCRIH.2153.C1_s_at	7538	ZFP36	Zinc finger protein 36, C3H type, homolog (mouse)
ADXCRIH.1510.C1_s_at	3638	INSIG1	Insulin induced gene 1
ADXCRIH.3127.C1_at	7112	TMPO	Thymopoietin
ADXCRIH.3127.C1_x_at	7112	TMPO	Thymopoietin
ADXCRIH.1533.C1_s_at	353	APRT	Adenine phosphoribosyltransferase
ADXCRIH.2536.C1_at	7203	CCT3	Chaperonin containing TCP1, subunit 3 (gamma)
ADXCRIH.2577.C1_s_at	56667	MUC13	Mucin 13, cell surface associated
ADXCRIH.2579.C1_at	60488	MRPS35	Mitochondrial ribosomal protein S35
ADXCRIH.2579.C1_x_at	60488	MRPS35	Mitochondrial ribosomal protein S35
ADXCRIH.1963.C1_s_at	80162	ATHL1	ATH1, acid trehalase-like 1 (yeast)
ADXCRIH.2211.C1_s_at	10083	USH1C	Usher syndrome 1C (autosomal recessive, severe)
ADXCRIH.994.C1_s_at	7112	TMPO	Thymopoietin
ADXCRIH.255.C1_at	3960	LGALS4	Lectin, galactoside-binding, soluble, 4 (galectin 4)
ADXCRIH.350.C2_s_at	6137	RPL13	Ribosomal protein L13
ADXCRIH.613.C1_at	23521	RPL13A	Ribosomal protein L13a
ADXCRIH.1445.C1_at	54458	PRR13	Proline rich 13
ADXCRIH.157.C4_s_at	3146	HMGB1	High-mobility group box 1
ADXCRIH.257.C1_at	7103	TSPAN8	Tetraspanin 8
ADXCRIHRC.3349.C1_s_at	23019	CNOT1	CCR4-NOT transcription complex, subunit 1
ADXCRIHRC.469.C1_s_at	29880	ALG5	Asparagine-linked glycosylation 5 homolog (S. cerevisiae, dolichyl-phosphate beta-glucosyltransferase)
ADXCRIHRC.3127.C1_at	7112	TMPO	Thymopoietin
ADXCRIHRC.2536.C1_at	7203	CCT3	Chaperonin containing TCP1, subunit 3 (gamma)
ADXCRIHRC.2579.C1_at	60488	MRPS35	Mitochondrial ribosomal protein S35
ADXCRIHRC.613.C1_x_at	23521	RPL13A	Ribosomal protein L13a

RDCR023_G12_at	7103	TSPAN8	Tetraspanin 8
RDCR023_G12_x_at	7103	TSPAN8	Tetraspanin 8
1_RDCR049_C08_at	7103	TSPAN8	Tetraspanin 8
RDCR490_C03_at	91860	CALML4	Calmodulin-like 4
RDCR490_C03_x_at	91860	CALML4	Calmodulin-like 4
ADXCRIHRC.2093.C1_s_at	100529241	HSPE1-MOB4	
ADXCRPD.7962.C1_s_at	6573	SLC19A1	Solute carrier family 19 (folate transporter), member 1
ADXCRPD.9220.C1_at	23082	PPRC1	Peroxisome proliferator-activated receptor gamma, coactivator-related 1
ADXCRPD.9220.C1_x_at	23082	PPRC1	Peroxisome proliferator-activated receptor gamma, coactivator-related 1
ADXCRPD.10475.C1_at	2877	GPX2	Glutathione peroxidase 2 (gastrointestinal)
ADXCRPD.18308.C1_at	9920	KBTBD11	Kelch repeat and BTB (POZ) domain containing 11
ADXCRPD.18376.C1_s_at	10083	USH1C	Usher syndrome 1C (autosomal recessive, severe)
ADXCRPD.8805.C1_at	400866	LINC00114	
ADXCRPD.10643.C1_at	7371	UCK2	Uridine-cytidine kinase 2
ADXCRPD.235.C1_at	NA	NA	
ADXCRPD.277.C1_x_at	11214	AKAP13	A kinase (PRKA) anchor protein 13
ADXCRPD.1914.C1_at	840	CASP7	Caspase 7, apoptosis-related cysteine peptidase
ADXCRPD.1914.C1_x_at	840	CASP7	Caspase 7, apoptosis-related cysteine peptidase
ADXCRPD.9879.C1_s_at	7371	UCK2	Uridine-cytidine kinase 2
ADXCRPD.500.C1_at	NA	NA	
ADXCRPD.13022.C1_s_at	27250	PDCD4	Programmed cell death 4 (neoplastic transformation inhibitor)
ADXCRPD.3556.C1_at	NA	NA	
ADXCRPD.3658.C1_at	NA	NA	
ADXCRPD.5046.C1_s_at	2729	GCLC	Glutamate-cysteine ligase, catalytic subunit
ADXCRPD.5097.C1_s_at	7371	UCK2	Uridine-cytidine kinase 2
ADXCRPD.3844.C1_x_at	80063	ATF7IP2	Activating transcription factor 7 interacting protein 2
ADXCRPD.13574.C1_at	10985	GCN1L1	GCN1 general control of amino-acid synthesis 1-like 1 (yeast)
ADXCRPD.13574.C1_s_at	10985	GCN1L1	GCN1 general control of amino-acid synthesis 1-like 1 (yeast)
ADXCRPD.12957.C1_at	7112	TMPO	Thymopoietin
ADXCRPD.4724.C1_s_at	144699	FBXL14	F-box and leucine-rich repeat protein 14
ADXCRPD.14415.C1_s_at	7371	UCK2	Uridine-cytidine kinase 2
ADXCRPD.15116.C1_at	7371	UCK2	Uridine-cytidine kinase 2
ADXCRPD.5551.C1_at	NA	NA	
ADXCRPD.5572.C1_s_at	27250	PDCD4	Programmed cell death 4 (neoplastic transformation inhibitor)
ADXCRPD.14599.C1_at	7371	UCK2	Uridine-cytidine kinase 2
ADXCRPD.14624.C1_at	100190939	TPT1-AS1	
ADXCRPD.6310.C1_s_at	23019	CNOT1	CCR4-NOT transcription complex, subunit 1
ADXCRAD_BX431388_s_at	91860	CALML4	Calmodulin-like 4
ADXCRAD_AJ708476_s_at	80162	ATHL1	ATH1, acid trehalase-like 1 (yeast)
ADXCRPD.15558.C1_s_at	79726	WDR59	WD repeat domain 59
ADXCRPD.15673.C1_at	11073	TOPBP1	Topoisomerase (DNA) II binding protein 1
ADXCRPD.6684.C1_s_at	1844	DUSP2	Dual specificity phosphatase 2
ADXCRPD.8111.C1_at	4609	MYC	V-myc myelocytomatosis viral oncogene homolog (avian)
ADXCRPD.16616.C1_at	5373	PMM2	Phosphomannomutase 2
ADXCRPD.16668.C1_at	23082	PPRC1	Peroxisome proliferator-activated receptor gamma, coactivator-related 1
ADXCRPD.10055.C1_at	7295	TXN	Thioredoxin
ADXCRPD.8360.C1_s_at	2729	GCLC	Glutamate-cysteine ligase, catalytic subunit
ADXCRPD.232.C2_s_at	8125	ANP32A	Acidic (leucine-rich) nuclear phosphoprotein 32 family, member A
ADXCRPDRC.18308.C1_at	NA	NA	
ADXCRPDRC.5097.C1_at	7371	UCK2	Uridine-cytidine kinase 2
ADXCRPDRC.5097.C1_x_at	7371	UCK2	Uridine-cytidine kinase 2
ADXCRPDRC.14624.C1_at	100190939	TPT1-AS1	
ADXCRPDRC.16668.C1_at	23082	PPRC1	Peroxisome proliferator-activated receptor gamma, coactivator-related 1
ADXCRPDRC.11127.C1_at	23590	PDSS1	Prenyl (decaprenyl) diphosphate synthase, subunit 1
ADXCRSS.Hs#S1228156_at	NA	NA	
ADXCRSS.Hs#S2874408_at	100128590	SLC8A1-AS1	
ADXCRSS.Hs#S1435288_at	100528062	ARMCX5-GPRASP2	
ADXCRSS.Hs#S11048289_at	29880	ALG5	Asparagine-linked glycosylation 5 homolog (S. cerevisiae, dolichyl-phosphate beta-glucosyltransferase)
ADXCRSS.Hs#S1861798_at	288	ANK3	Ankyrin 3, node of Ranvier (ankyrin G)
ADXCRSS.Hs#S1923985_at	51019	CCDC53	Coiled-coil domain containing 53
ADXCRSS.Hs#S1924358_at	27086	FOXP1	Forkhead box P1
ADXCRSS.Hs#S2978009_at	NA	NA	
ADXCRSS.Hs#S2978009_x_at	51427	ZNF107	Zinc finger protein 107
ADXCRSS.Hs#S2979085_at	2963	GTF2F2	General transcription factor IIF, polypeptide 2, 30kDa
ADXCRSS.Hs#S2988186_at	54982	CLN6	
ADXCRSS.Hs#S3017795_at	NA	NA	
ADXCRSS.Hs#S3017613_at	840	CASP7	Caspase 7, apoptosis-related cysteine peptidase
ADXCRSS.Hs#S3746017_at	29068	ZBTB44	Zinc finger and BTB domain containing 44
ADXCRSS.Hs#S3746017_x_at	29068	ZBTB44	Zinc finger and BTB domain containing 44
ADXCRSS.Hs#S3736200_at	2078	ERG	V-ets erythroblastosis virus E26 oncogene homolog (avian)
ADXCRSS.Hs#S3738574_at	2114	ETS2	V-ets erythroblastosis virus E26 oncogene homolog 2 (avian)
ADXCRSS.Hs#S854806_at	26147	PHF19	PHD finger protein 19
ADXCRIH.572.C1_s_at	1933	EEF1B2	Eukaryotic translation elongation factor 1 beta 2
ADXCRIH.572.C3_at	1933	EEF1B2	Eukaryotic translation elongation factor 1 beta 2
ADXCRIH.1252.C1_at	1365	CLDN3	Claudin 3
ADXCRIH.1252.C1_x_at	1365	CLDN3	Claudin 3
ADXCRIH.2093.C1_at	100529241	HSPE1-MOB4	
ADXCRIH.2093.C1_s_at	100529241	HSPE1-MOB4	
ADXCRIH.2093.C2_at	3336	HSPE1	Heat shock 10kDa protein 1 (chaperonin 10)
ADXCRIH.2093.C2_x_at	3336	HSPE1	Heat shock 10kDa protein 1 (chaperonin 10)
ADXCRIH.2576.C1_s_at	2114	ETS2	V-ets erythroblastosis virus E26 oncogene homolog 2 (avian)
ADXCRIH.2576.C2_at	2114	ETS2	V-ets erythroblastosis virus E26 oncogene homolog 2 (avian)
ADXCRPD.724.C1_at	5557	PRIM1	Primase, DNA, polypeptide 1 (49kDa)
ADXCRPD.724.C2_at	5557	PRIM1	Primase, DNA, polypeptide 1 (49kDa)

ADXCRPD.10538.C2_at	23085	ERC1	ELKS/RAB6-interacting/CAST family member 1
ADXCRPD.11257.C1_at	288	ANK3	Ankyrin 3, node of Ranvier (ankyrin G)
ADXCRPD.12126.C1_at	5801	PTPRR	Protein tyrosine phosphatase, receptor type, R
ADXCRPD.12126.C1_s_at	23019	CNOT1	CCR4-NOT transcription complex, subunit 1
ADXCRPD.12126.C2_at	NA	NA	
ADXCRPD.12126.C2_s_at	23019	CNOT1	CCR4-NOT transcription complex, subunit 1
ADXCRAD_DN602116_s_at	3326	HSP90AB1	Heat shock protein 90kDa alpha (cytosolic), class B member 1
ADXCRAD_BQ928122_at	54982	CLN6	
ADXCRAD_BQ928122_x_at	54982	CLN6	
ADXCRAD_AL547157_s_at	23520	ANP32C	Acidic (leucine-rich) nuclear phosphoprotein 32 family, member C
ADXCRAD_AL547157_x_at	8125	ANP32A	Acidic (leucine-rich) nuclear phosphoprotein 32 family, member A
ADXCRAD_BU191559_at	3638	INSIG1	Insulin induced gene 1
ADXCRAD_BU570382_at	3638	INSIG1	Insulin induced gene 1
ADXCRAD_BU570382_x_at	3638	INSIG1	Insulin induced gene 1
ADXCRAD_CD511474_at	27010	TPK1	Thiamin pyrophosphokinase 1
ADXCRAD_CD511474_s_at	27010	TPK1	Thiamin pyrophosphokinase 1
ADXCRAD_CD511474_x_at	27010	TPK1	Thiamin pyrophosphokinase 1
ADXCRAD_CA454569_at	NA	NA	
ADXCRAD_CN401906_s_at	3146	HMGB1	High-mobility group box 1
ADXCRAD_BE390153_at	5373	PMM2	Phosphomannomutase 2
ADXCRAD_BG571275_at	2960	GTF2E1	General transcription factor IIE, polypeptide 1, alpha 56kDa
ADXCRAD_D11859_s_at	1992	SERPINB1	Serpin peptidase inhibitor, clade B (ovalbumin), member 1
ADXCRAD_BQ188744_s_at	2194	FASN	Fatty acid synthase
ADXCRAD_BE544684_at	92482	BBIP1	
ADXCRAD_BE544684_x_at	92482	BBIP1	
ADXCRAD_BP384660_s_at	6472	SHMT2	Serine hydroxymethyltransferase 2 (mitochondrial)
ADXCRAD_BX429991_at	NA	NA	
ADXCRAD_BQ890025_at	NA	NA	
ADXCRAD_BQ890025_x_at	NA	NA	
ADXCRAD_BX092880_at	288	ANK3	Ankyrin 3, node of Ranvier (ankyrin G)
ADXCRAD_BM313583_s_at	3329	HSPD1	Heat shock 60kDa protein 1 (chaperonin)
ADXCRAD_CR745173_s_at	27010	TPK1	Thiamin pyrophosphokinase 1
ADXCRAD_AA393120_at	79726	WDR59	WD repeat domain 59
ADXCRAD_DN601918_x_at	3146	HMGB1	High-mobility group box 1
ADXCRAD_AL541939_s_at	3638	INSIG1	Insulin induced gene 1
ADXCRAD_CX757597_at	NA	NA	
ADXCRAD_BG756941_at	100507377	LOC100507377	
ADXCRAD_BM907731_at	NA	NA	
ADXCRAD_BX482009_s_at	7112	TMPO	Thymopoietin
ADXCRAD_BM550622_at	NA	NA	
ADXCRAD_BM550622_x_at	27250	PDCD4	Programmed cell death 4 (neoplastic transformation inhibitor)
ADXCRAD_CX788770_at	6472	SHMT2	Serine hydroxymethyltransferase 2 (mitochondrial)
ADXCRAD_CX788770_s_at	6472	SHMT2	Serine hydroxymethyltransferase 2 (mitochondrial)
ADXCRAD_CX785467_at	10921	RNPS1	RNA binding protein S1, serine-rich domain
ADXCRAD_CX785467_x_at	10921	RNPS1	RNA binding protein S1, serine-rich domain
ADXCRAD_BU189824_at	471	ATIC	5-aminoimidazole-4-carboxamide ribonucleotide formyltransferase/IMP cyclohydrolase
ADXCRAD_CN349881_s_at	8125	ANP32A	Acidic (leucine-rich) nuclear phosphoprotein 32 family, member A
ADXCRAD_BM978758_at	2936	GSR	Glutathione reductase
ADXCRAD_NM_206999_at	23019	CNOT1	CCR4-NOT transcription complex, subunit 1
ADXCRAD_NM_004843_at	56776	FMN2	Formin 2
ADXCRAD_NM_014867_at	9920	KBTBD11	Kelch repeat and BTB (POZ) domain containing 11
ADXCRAD_BC043171_at	27250	PDCD4	Programmed cell death 4 (neoplastic transformation inhibitor)
ADXCRAD_BC043171_x_at	27250	PDCD4	Programmed cell death 4 (neoplastic transformation inhibitor)
ADXCRAD_T67821_at	8125	ANP32A	Acidic (leucine-rich) nuclear phosphoprotein 32 family, member A
ADXCRAD_T67821_x_at	8125	ANP32A	Acidic (leucine-rich) nuclear phosphoprotein 32 family, member A
ADXCRAD_AI632259_x_at	25942	SIN3A	SIN3 homolog A, transcription regulator (yeast)
ADXCRAD_W52010_s_at	1992	SERPINB1	Serpin peptidase inhibitor, clade B (ovalbumin), member 1
ADXCRAD_AI348378_at	91860	CALML4	Calmodulin-like 4
ADXCRAD_AV646177_at	2114	ETS2	V-ets erythroblastosis virus E26 oncogene homolog 2 (avian)
ADXCRAD_NM_025034_at	10083	USH1C	Usher syndrome 1C (autosomal recessive, severe)
ADXCRAD_AW674195_at	3329	HSPD1	Heat shock 60kDa protein 1 (chaperonin)
ADXCRAD_AW674195_x_at	3329	HSPD1	Heat shock 60kDa protein 1 (chaperonin)
ADXCRAD_NM_006711_at	147664	ERVV-1	
ADXCRAD_AK023838_x_at	4249	MGAT5	Mannosyl (alpha-1,6-)-glycoprotein beta-1,6-N-acetyl-glucosaminyltransferase
ADXCRAD_AI554467_at	6137	RPL13	Ribosomal protein L13
ADXCRAD_AI554467_x_at	6137	RPL13	Ribosomal protein L13
ADXCRAD_AW025529_at	91860	CALML4	Calmodulin-like 4

S2 Table part B

probeset_id	entrez_id	hgnc_symbol	Description
ADXCRAG_AB028971_s_at	22848	AAK1	AP2 associated kinase 1
ADXCRAG_AC007401_at	51232	CRIM1	Cysteine rich transmembrane BMP regulator 1 (chordin-like)
ADXCRAD_CA396083_s_at	6840	SVIL	Supervillin
ADXCRAG_AF117949_s_at	NA	NA	
ADXCRAG_AF118076_at	1063	CENPF	Centromere protein F, 350/400ka (mitosin)
ADXCRAG_AF118076_x_at	1063	CENPF	Centromere protein F, 350/400ka (mitosin)
ADXCRAG_AF138861_s_at	1112	FOXN3	Forkhead box N3
ADXCRAG_AF142421_s_at	9444	QKI	Quaking homolog, KH domain RNA binding (mouse)
ADXCRAG_AF168681_x_at	129684	CNTNAP5	Contactin associated protein-like 5
ADXCRAG_AF177394_s_at	22943	DKK1	Dickkopf homolog 1 (Xenopus laevis)
ADXCRPD.6828.C1_s_at	55700	MAP7D1	MAP7 domain containing 1
ADXCRAG_AF383172_s_at	23032	USP33	Ubiquitin specific peptidase 33

ADXCRAG_AF392444_s_at	26031	OSBPL3	Oxysterol binding protein-like 3
ADXCRAG_AF480883_at	9499	MYOT	Myotilin
ADXCRAG_AY309920_s_at	10184	LHFPL2	Lipoma HMGIC fusion partner-like 2
ADXCRAG_CD251162_s_at	11328	FKBP9	FK506 binding protein 9, 63 kDa
ADXCRAG_BC008726_s_at	9943	OXSR1	Oxidative-stress responsive 1
ADXCRAG_BC011827_s_at	4478	MSN	Moesin
ADXCRAG_BC011987_s_at	87	ACTN1	Actinin, alpha 1
ADXCRAG_BC023584_s_at	24140	FTSJ1	FtsJ homolog 1 (E. coli)
ADXCRAD_AL046837_s_at	7431	VIM	Vimentin
ADXCRAG_BC036087_s_at	5813	PURA	Purine-rich element binding protein A
ADXCRAG_BC065036_s_at	9448	MAP4K4	Mitogen-activated protein kinase kinase kinase kinase 4
ADXCRAD_CB217549_s_at	7026	NR2F2	Nuclear receptor subfamily 2, group F, member 2
ADXCRAG_BX648297_s_at	4026	LPP	LIM domain containing preferred translocation partner in lipoma
ADXCRAG_D21254_s_at	1009	CDH11	Cadherin 11, type 2, OB-cadherin (osteoblast)
ADXCRAD_BM789115_s_at	9697	TRAM2	Translocation associated membrane protein 2
ADXCRAG_M69066_s_at	4478	MSN	Moesin
ADXCRAD_BP396775_s_at	966	CD59	CD59 molecule, complement regulatory protein
ADXCRAG_NM_001001549_s_at	2887	GRB10	Growth factor receptor-bound protein 10
ADXCRAD_CV574618_s_at	4015	LOX	Lysyl oxidase
ADXCRAG_NM_002318_s_at	4017	LOXL2	Lysyl oxidase-like 2
ADXCRAG_NM_002526_at	4907	NT5E	5'-nucleotidase, ecto (CD73)
ADXCRAG_NM_002526_s_at	4907	NT5E	5'-nucleotidase, ecto (CD73)
ADXCRAD_CF130280_s_at	5708	PSMD2	Proteasome (prosome, macropain) 26S subunit, non-ATPase, 2
ADXCRAD_CD678016_s_at	8613	PPAP2B	Phosphatidic acid phosphatase type 2B
ADXCRAG_NM_005578_at	4026	LPP	LIM domain containing preferred translocation partner in lipoma
ADXCRAG_NM_014911_s_at	22848	AAK1	AP2 associated kinase 1
ADXCRAG_NM_133631_s_at	6091	ROBO1	Roundabout, axon guidance receptor, homolog 1 (Drosophila)
ADXCRAD_CN265168_s_at	3572	IL6ST	Interleukin 6 signal transducer (gp130, oncostatin M receptor)
ADXCRAD_BM931336_s_at	9444	QKI	Quaking homolog, KH domain RNA binding (mouse)
ADXCRAG_U67093_s_at	472	ATM	Ataxia telangiectasia mutated
ADXCRAG_U68723_at	1112	FOXN3	Forkhead box N3
ADXCRAD_CR735795_s_at	1112	FOXN3	Forkhead box N3
ADXCRAG_X03348_s_at	2908	NR3C1	Nuclear receptor subfamily 3, group C, member 1 (glucocorticoid receptor)
ADXCRAG_XM_371497_x_at	22848	AAK1	AP2 associated kinase 1
ADXCRAG_Z70660_at	5796	PTPRK	Protein tyrosine phosphatase, receptor type, K
ADXCRAD_CN347079_s_at	960	CD44	CD44 molecule (Indian blood group)
ADXCRIH.653.C1_s_at	4082	MARCKS	Myristoylated alanine-rich protein kinase C substrate
ADXCRIH.2305.C1_at	5813	PURA	Purine-rich element binding protein A
ADXCRIH.2305.C1_x_at	5813	PURA	Purine-rich element binding protein A
ADXCRAD_BG621095_s_at	51232	CRIM1	Cysteine rich transmembrane BMP regulator 1 (chordin-like)
ADXCRAD_CX867144_s_at	8682	PEA15	Phosphoprotein enriched in astrocytes 15
ADXCRIH.2427.C1_s_at	1266	CNN3	Calponin 3, acidic
ADXCRIH.436.C1_at	7431	VIM	Vimentin
ADXCRIH.3823.C1_at	6840	SVIL	Supervillin
ADXCRIH.3832.C1_s_at	56005	C19orf10	Chromosome 19 open reading frame 10
ADXCRIH.2899.C1_at	3956	LGALS1	Lectin, galactoside-binding, soluble, 1 (galectin 1)
ADXCRIH.91.C1_at	5796	PTPRK	Protein tyrosine phosphatase, receptor type, K
ADXCRIH.2503.C1_at	10213	PSMD14	Proteasome (prosome, macropain) 26S subunit, non-ATPase, 14
ADXCRIH.1579.C1_at	3491	CYR61	Cysteine-rich, angiogenic inducer, 61
ADXCRAD_CX788634_s_at	813	CALU	Calumenin
ADXCRAD_BX365807_s_at	51474	LIMA1	LIM domain and actin binding 1
ADXCRIH.1264.C1_s_at	5621	PRNP	Prion protein (p27-30) (Creutzfeldt-Jakob disease, Gerstmann-Strausler-Scheinker syndrome, fatal familial insomnia)
ADXCRIH.243.C1_at	9805	SCRN1	Secernin 1
ADXCRIH.243.C1_s_at	9805	SCRN1	Secernin 1
ADXCRIH.335.C3_s_at	989	SEPT7	Septin 7
ADXCRIH.194.C3_at	203068	TUBB	Tubulin, beta
ADXCRIH.194.C3_x_at	203068	TUBB	Tubulin, beta
ADXCRIH.1095.C2_at	857	CAV1	Caveolin 1, caveolae protein, 22kDa
ADXCRIH.476.C2_at	10049	DNAJB6	DnaJ (Hsp40) homolog, subfamily B, member 6
ADXCRIH.716.C2_at	3725	JUN	Jun oncogene
ADXCRIH.716.C2_s_at	3725	JUN	Jun oncogene
ADXCRIH.1058.C2_s_at	5110	PCMT1	Protein-L-isoaspartate (D-aspartate) O-methyltransferase
ADXCRIHRC.653.C1_at	4082	MARCKS	Myristoylated alanine-rich protein kinase C substrate
ADXCRIHRC.653.C1_x_at	4082	MARCKS	Myristoylated alanine-rich protein kinase C substrate
ADXCRIHRC.2305.C1_s_at	5813	PURA	Purine-rich element binding protein A
ADXCRAD_CX873552_s_at	7431	VIM	Vimentin
ADXCRIHRC.3823.C1_at	6840	SVIL	Supervillin
ADXCRAD_CN402078_s_at	6840	SVIL	Supervillin
ADXCRIHRC.2899.C1_at	3956	LGALS1	Lectin, galactoside-binding, soluble, 1 (galectin 1)
ADXCRIHRC.91.C1_s_at	5796	PTPRK	Protein tyrosine phosphatase, receptor type, K
RDCR156_A02_at	10369	CACNG2	Calcium channel, voltage-dependent, gamma subunit 2
RDCR156_A02_x_at	10369	CACNG2	Calcium channel, voltage-dependent, gamma subunit 2
RDCR181_H11_at	286006	C7orf53	Chromosome 7 open reading frame 53
RDCR181_H11_x_at	286006	C7orf53	Chromosome 7 open reading frame 53
RDCR166_G12_at	NA	NA	
RDCR166_G12_s_at	10049	DNAJB6	DnaJ (Hsp40) homolog, subfamily B, member 6
RDCR092_B07_s_at	22848	AAK1	AP2 associated kinase 1
RDCR460_E08_at	5796	PTPRK	Protein tyrosine phosphatase, receptor type, K
ADXCRIHRC.1441.C1_s_at	960	CD44	CD44 molecule (Indian blood group)
ADXCRPD.16964.C1_at	2316	FLNA	Filamin A, alpha (actin binding protein 280)
ADXCRPD.16965.C1_at	NA	NA	
ADXCRPD.1395.C1_at	9448	MAP4K4	Mitogen-activated protein kinase kinase kinase kinase 4
ADXCRPD.17623.C1_s_at	9448	MAP4K4	Mitogen-activated protein kinase kinase kinase kinase 4
ADXCRPD.8652.C1_at	1112	FOXN3	Forkhead box N3

ADXCRPD.8667.C1_s_at	11031	RAB31	RAB31, member RAS oncogene family
ADXCRPD.11024.C1_s_at	2273	FHL1	Four and a half LIM domains 1
ADXCRPD.2065.C1_at	NA	NA	
ADXCRPD.10462.C1_s_at	1112	FOXN3	Forkhead box N3
ADXCRPD.17734.C1_at	439921	MXRA7	Matrix-remodelling associated 7
ADXCRPD.8765.C1_at	1112	FOXN3	Forkhead box N3
ADXCRPD.11104.C1_at	5796	PTPRK	Protein tyrosine phosphatase, receptor type, K
ADXCRPD.8770.C1_at	2534	FYN	FYN oncogene related to SRC, FGR, YES
ADXCRPD.11183.C1_at	4026	LPP	LIM domain containing preferred translocation partner in lipoma
ADXCRPD.1565.C1_at	54741	LEPROT	
ADXCRPD.10589.C1_at	3491	CYR61	Cysteine-rich, angiogenic inducer, 61
ADXCRPD.1656.C1_s_at	1063	CENPF	Centromere protein F, 350/400ka (mitosin)
ADXCRPD.11316.C1_at	390	RND3	Rho family GTPase 3
ADXCRPD.2332.C1_at	NA	NA	
ADXCRPD.10702.C1_s_at	284119	PTRF	Polymerase I and transcript release factor
ADXCRPD.129.C1_at	NA	NA	
ADXCRPD.12020.C1_s_at	6840	SVIL	Supervillin
ADXCRPD.3034.C1_at	2316	FLNA	Filamin A, alpha (actin binding protein 280)
ADXCRPD.3071.C1_s_at	472	ATM	Ataxia telangiectasia mutated
ADXCRPD.2471.C1_at	11031	RAB31	RAB31, member RAS oncogene family
ADXCRPD.11491.C1_at	493	ATP2B4	ATPase, Ca++ transporting, plasma membrane 4
ADXCRPD.12156.C1_at	1112	FOXN3	Forkhead box N3
ADXCRPD.12156.C1_s_at	1112	FOXN3	Forkhead box N3
ADXCRPD.2523.C1_s_at	7030	TFE3	Transcription factor binding to IGHM enhancer 3
ADXCRPD.2526.C1_at	824	CAPN2	Calpain 2, (m/II) large subunit
ADXCRPD.10919.C1_at	5796	PTPRK	Protein tyrosine phosphatase, receptor type, K
ADXCRPD.10919.C1_x_at	5796	PTPRK	Protein tyrosine phosphatase, receptor type, K
ADXCRPD.11577.C1_s_at	960	CD44	CD44 molecule (Indian blood group)
ADXCRPD.1932.C1_s_at	9444	QKI	Quaking homolog, KH domain RNA binding (mouse)
ADXCRPD.1938.C1_s_at	11031	RAB31	RAB31, member RAS oncogene family
ADXCRPD.3227.C1_at	2316	FLNA	Filamin A, alpha (actin binding protein 280)
ADXCRPD.9920.C1_at	6840	SVIL	Supervillin
ADXCRPD.9976.C1_at	824	CAPN2	Calpain 2, (m/II) large subunit
ADXCRPD.2725.C1_at	6617	SNAPC1	Small nuclear RNA activating complex, polypeptide 1, 43kDa
ADXCRPD.558.C1_at	813	CALU	Calumenin
ADXCRAD_CN403921_s_at	813	CALU	Calumenin
ADXCRPD.2801.C1_at	26031	OSBPL3	Oxysterol binding protein-like 3
ADXCRPD.11859.C1_at	3572	IL6ST	Interleukin 6 signal transducer (gp130, oncostatin M receptor)
ADXCRPD.2854.C1_x_at	9669	EIF5B	Eukaryotic translation initiation factor 5B
ADXCRPD.11873.C1_at	51232	CRIM1	Cysteine rich transmembrane BMP regulator 1 (chordin-like)
ADXCRPD.11873.C1_s_at	51232	CRIM1	Cysteine rich transmembrane BMP regulator 1 (chordin-like)
ADXCRAD_NM_021913_s_at	558	AXL	AXL receptor tyrosine kinase
ADXCRPD.3501.C1_s_at	595	CCND1	Cyclin D1
ADXCRAD_BM551840_x_at	595	CCND1	Cyclin D1
ADXCRPD.13194.C1_at	5796	PTPRK	Protein tyrosine phosphatase, receptor type, K
ADXCRPD.13198.C1_at	5796	PTPRK	Protein tyrosine phosphatase, receptor type, K
ADXCRPD.2979.C1_at	26031	OSBPL3	Oxysterol binding protein-like 3
ADXCRPD.2985.C1_at	9448	MAP4K4	Mitogen-activated protein kinase kinase kinase kinase 4
ADXCRPD.737.C1_s_at	11004	KIF2C	Kinesin family member 2C
ADXCRPD.13338.C1_at	2316	FLNA	Filamin A, alpha (actin binding protein 280)
ADXCRPD.4372.C1_s_at	2534	FYN	FYN oncogene related to SRC, FGR, YES
ADXCRPD.5015.C1_s_at	5376	PMP22	Peripheral myelin protein 22
ADXCRPD.13412.C1_s_at	8613	PPAP2B	Phosphatidic acid phosphatase type 2B
ADXCRPD.14082.C1_at	5796	PTPRK	Protein tyrosine phosphatase, receptor type, K
ADXCRPD.4423.C1_at	4082	MARCKS	Myristoylated alanine-rich protein kinase C substrate
ADXCRPD.4423.C1_x_at	4082	MARCKS	Myristoylated alanine-rich protein kinase C substrate
ADXCRPD.14111.C1_at	10049	DNAJB6	DnaJ (Hsp40) homolog, subfamily B, member 6
ADXCRPD.14170.C1_at	7030	TFE3	Transcription factor binding to IGHM enhancer 3
ADXCRPD.12932.C1_at	26031	OSBPL3	Oxysterol binding protein-like 3
ADXCRPD.3926.C1_at	NA	NA	
ADXCRPD.4590.C1_at	4017	LOXL2	Lysyl oxidase-like 2
ADXCRPD.3980.C1_s_at	595	CCND1	Cyclin D1
ADXCRPD.5241.C1_at	4907	NT5E	5'-nucleotidase, ecto (CD73)
ADXCRPD.14289.C1_at	8682	PEA15	Phosphoprotein enriched in astrocytes 15
ADXCRPD.14289.C1_s_at	8682	PEA15	Phosphoprotein enriched in astrocytes 15
ADXCRPD.14289.C1_x_at	8682	PEA15	Phosphoprotein enriched in astrocytes 15
ADXCRPD.13648.C1_at	3572	IL6ST	Interleukin 6 signal transducer (gp130, oncostatin M receptor)
ADXCRPD.13648.C1_x_at	3572	IL6ST	Interleukin 6 signal transducer (gp130, oncostatin M receptor)
ADXCRPD.4641.C1_s_at	3475	IFRD1	Interferon-related developmental regulator 1
ADXCRPD.4673.C1_at	7026	NR2F2	Nuclear receptor subfamily 2, group F, member 2
ADXCRPD.5301.C1_s_at	966	CD59	CD59 molecule, complement regulatory protein
ADXCRPD.5328.C1_s_at	4026	LPP	LIM domain containing preferred translocation partner in lipoma
ADXCRPD.6080.C1_at	NA	NA	
ADXCRPD.14481.C1_s_at	3572	IL6ST	Interleukin 6 signal transducer (gp130, oncostatin M receptor)
ADXCRPD.13832.C1_s_at	9130	FAM50A	Family with sequence similarity 50, member A
ADXCRPD.5554.C1_at	10184	LHFPL2	Lipoma HMGIC fusion partner-like 2
ADXCRPD.13939.C1_at	595	CCND1	Cyclin D1
ADXCRPD.14604.C1_at	4026	LPP	LIM domain containing preferred translocation partner in lipoma
ADXCRPD.15300.C1_at	2887	GRB10	Growth factor receptor-bound protein 10
ADXCRPD.6370.C1_at	9130	FAM50A	Family with sequence similarity 50, member A
ADXCRPD.6370.C1_x_at	9130	FAM50A	Family with sequence similarity 50, member A
ADXCRPD.15392.C1_at	966	CD59	CD59 molecule, complement regulatory protein
ADXCRPD.16054.C1_at	595	CCND1	Cyclin D1
ADXCRPD.16135.C1_at	11328	FKBP9	FK506 binding protein 9, 63 kDa

ADXCRPD.16135.C1_x_at	360132	FKBP9L	FK506 binding protein 9-like
ADXCRPD.14974.C1_at	472	ATM	Ataxia telangiectasia mutated
ADXCRPD.15612.C1_at	2316	FLNA	Filamin A, alpha (actin binding protein 280)
ADXCRPD.15616.C1_at	51232	CRIM1	Cysteine rich transmembrane BMP regulator 1 (chordin-like)
ADXCRPD.7382.C1_at	9263	STK17A	Serine/threonine kinase 17a
ADXCRPD.17056.C1_at	1063	CENPF	Centromere protein F, 350/400ka (mitosin)
ADXCRPD.6814.C1_s_at	5796	PTPRK	Protein tyrosine phosphatase, receptor type, K
ADXCRPD.15960.C1_at	595	CCND1	Cyclin D1
ADXCRPD.16626.C1_s_at	4026	LPP	LIM domain containing preferred translocation partner in lipoma
ADXCRPD.8271.C1_at	4082	MARCKS	Myristoylated alanine-rich protein kinase C substrate
ADXCRPD.8271.C1_x_at	4082	MARCKS	Myristoylated alanine-rich protein kinase C substrate
ADXCRPD.16635.C1_at	4026	LPP	LIM domain containing preferred translocation partner in lipoma
ADXCRPD.7689.C1_at	25907	TMEM158	Transmembrane protein 158
ADXCRPD.7689.C1_x_at	25907	TMEM158	Transmembrane protein 158
ADXCRPD.10038.C1_at	2273	FHL1	Four and a half LIM domains 1
ADXCRPD.1038.C1_s_at	7077	TIMP2	TIMP metallopeptidase inhibitor 2
ADXCRPD.10094.C1_at	493	ATP2B4	ATPase, Ca++ transporting, plasma membrane 4
ADXCRPD.10094.C1_x_at	493	ATP2B4	ATPase, Ca++ transporting, plasma membrane 4
ADXCRPD.1098.C1_s_at	1266	CNN3	Calponin 3, acidic
ADXCRPD.17316.C1_at	22848	AAK1	AP2 associated kinase 1
ADXCRAD_BP325154_s_at	2273	FHL1	Four and a half LIM domains 1
ADXCRPD.10116.C1_s_at	9943	OXSR1	Oxidative-stress responsive 1
ADXCRPD.10169.C1_at	2316	FLNA	Filamin A, alpha (actin binding protein 280)
ADXCRPD.1153.C1_s_at	390	RND3	Rho family GTPase 3
ADXCRPD.1189.C1_s_at	28951	TRIB2	Tribbles homolog 2 (Drosophila)
ADXCRPD.9098.C1_at	4026	LPP	LIM domain containing preferred translocation partner in lipoma
ADXCRPD.1261.C1_s_at	857	CAV1	Caveolin 1, caveolae protein, 22kDa
ADXCRPD.18131.C1_s_at	966	CD59	CD59 molecule, complement regulatory protein
ADXCRPD.17514.C1_at	7476	WNT7A	Wingless-type MMTV integration site family, member 7A
ADXCRPD.17514.C1_x_at	9444	QKI	Quaking homolog, KH domain RNA binding (mouse)
ADXCRPD.2301.C1_at	9805	SCRN1	Secernin 1
ADXCRPD.6630.C1_s_at	824	CAPN2	Calpain 2, (m/II) large subunit
ADXCRPD.3150.C3_at	2316	FLNA	Filamin A, alpha (actin binding protein 280)
ADXCRPD.3397.C1_at	7070	THY1	Thy-1 cell surface antigen
ADXCRPD.177.C1_s_at	989	SEPT7	Septin 7
ADXCRPD.4345.C1_at	10981	RAB32	RAB32, member RAS oncogene family
ADXCRPD.4345.C1_s_at	10981	RAB32	RAB32, member RAS oncogene family
ADXCRPD.2741.C1_at	NA	NA	
ADXCRPD.362.C1_at	7068	THRB	Thyroid hormone receptor, beta (erythroblastic leukemia viral (v-erb-a) oncogene homolog 2, avian)
ADXCRAD_CN309928_s_at	64175	LEPRE1	Leucine proline-enriched proteoglycan (leprecan) 1
ADXCRPD.3976.C1_s_at	2014	EMP3	Epithelial membrane protein 3
ADXCRPD.2246.C1_x_at	284119	PTRF	Polymerase I and transcript release factor
ADXCRPD.3075.C1_s_at	11328	FKBP9	FK506 binding protein 9, 63 kDa
ADXCRPDRC.1395.C1_s_at	9448	MAP4K4	Mitogen-activated protein kinase kinase kinase kinase 4
ADXCRPDRC.8652.C1_s_at	1112	FOXN3	Forkhead box N3
ADXCRPDRC.8765.C1_s_at	1112	FOXN3	Forkhead box N3
ADXCRPDRC.1565.C1_s_at	3953	LEPR	Leptin receptor
ADXCRPDRC.12156.C1_at	1112	FOXN3	Forkhead box N3
ADXCRPDRC.3227.C1_at	2316	FLNA	Filamin A, alpha (actin binding protein 280)
ADXCRPDRC.9920.C1_at	NA	NA	
ADXCRPDRC.9920.C1_s_at	6840	SVIL	Supervillin
ADXCRPDRC.9976.C1_at	824	CAPN2	Calpain 2, (m/II) large subunit
ADXCRPDRC.2801.C1_at	NA	NA	
ADXCRPDRC.2801.C1_s_at	26031	OSBPL3	Oxysterol binding protein-like 3
ADXCRPDRC.11859.C1_s_at	3572	IL6ST	Interleukin 6 signal transducer (gp130, oncostatin M receptor)
ADXCRPDRC.2979.C1_s_at	26031	OSBPL3	Oxysterol binding protein-like 3
ADXCRPDRC.2985.C1_s_at	9448	MAP4K4	Mitogen-activated protein kinase kinase kinase kinase 4
ADXCRPDRC.14082.C1_s_at	5796	PTPRK	Protein tyrosine phosphatase, receptor type, K
ADXCRPDRC.4423.C1_s_at	4082	MARCKS	Myristoylated alanine-rich protein kinase C substrate
ADXCRPDRC.14111.C1_at	NA	NA	
ADXCRPDRC.14111.C1_s_at	10049	DNAJB6	DnaJ (Hsp40) homolog, subfamily B, member 6
ADXCRPDRC.4590.C1_s_at	4017	LOXL2	Lysyl oxidase-like 2
ADXCRPDRC.4673.C1_s_at	7026	NR2F2	Nuclear receptor subfamily 2, group F, member 2
ADXCRPDRC.5554.C1_at	NA	NA	
ADXCRPDRC.6370.C1_at	9130	FAM50A	Family with sequence similarity 50, member A
ADXCRPDRC.15392.C1_at	4299	AFF1	AF4/FMR2 family, member 1
ADXCRPDRC.15392.C1_s_at	966	CD59	CD59 molecule, complement regulatory protein
ADXCRPDRC.16054.C1_at	595	CCND1	Cyclin D1
ADXCRPDRC.14974.C1_s_at	472	ATM	Ataxia telangiectasia mutated
ADXCRPDRC.15616.C1_s_at	51232	CRIM1	Cysteine rich transmembrane BMP regulator 1 (chordin-like)
ADXCRPDRC.8271.C1_s_at	4082	MARCKS	Myristoylated alanine-rich protein kinase C substrate
ADXCRPDRC.7689.C1_s_at	25907	TMEM158	Transmembrane protein 158
ADXCRPDRC.10038.C1_at	2273	FHL1	Four and a half LIM domains 1
ADXCRPDRC.17316.C1_at	22848	AAK1	AP2 associated kinase 1
ADXCRPDRC.9098.C1_s_at	4026	LPP	LIM domain containing preferred translocation partner in lipoma
ADXCRPDRC.3805.C1_s_at	3725	JUN	Jun oncogene
ADXCRAD_CK001870_at	NA	NA	
ADXCRAD_CK001870_s_at	9805	SCRN1	Secernin 1
ADXCRPDRC.3150.C3_s_at	2316	FLNA	Filamin A, alpha (actin binding protein 280)
ADXCRPDRC.3397.C1_s_at	7070	THY1	Thy-1 cell surface antigen
ADXCRPDRC.16964.C1_at	2316	FLNA	Filamin A, alpha (actin binding protein 280)
ADXCRPDRC.5728.C1_s_at	493	ATP2B4	ATPase, Ca++ transporting, plasma membrane 4
ADXCRSS.Hs#S1294625_at	4026	LPP	LIM domain containing preferred translocation partner in lipoma
ADXCRSS.Hs#S1299958_at	4907	NT5E	5'-nucleotidase, ecto (CD73)

ADXCRSS.Hs#S1805720_s_at	51232	CRIM1	Cysteine rich transmembrane BMP regulator 1 (chordin-like)
ADXCRSS.Hs#S1228681_at	960	CD44	CD44 molecule (Indian blood group)
ADXCRSS.Hs#S1449485_at	10184	LHFPL2	Lipoma HMGIC fusion partner-like 2
ADXCRSS.Hs#S11049845_at	148823	GCSAML	
ADXCRSS.Hs#S11049845_x_at	148823	GCSAML	
ADXCRSS.Hs#S1914330_at	51474	LIMA1	LIM domain and actin binding 1
ADXCRSS.Hs#S1914581_at	989	SEPT7	Septin 7
ADXCRSS.Hs#S1921365_at	10049	DNAJB6	DnaJ (Hsp40) homolog, subfamily B, member 6
ADXCRSS.Hs#S1921392_at	9448	MAP4K4	Mitogen-activated protein kinase kinase kinase kinase 4
ADXCRSS.Hs#S1921392_x_at	9448	MAP4K4	Mitogen-activated protein kinase kinase kinase kinase 4
ADXCRSS.Hs#S1921588_at	4026	LPP	LIM domain containing preferred translocation partner in lipoma
ADXCRSS.Hs#S3738155_at	51474	LIMA1	LIM domain and actin binding 1
ADXCRSS.Hs#S3738155_x_at	51474	LIMA1	LIM domain and actin binding 1
ADXCRSS.Hs#S2732094_at	4026	LPP	LIM domain containing preferred translocation partner in lipoma
ADXCRSS.Hs#S1908923_at	4026	LPP	LIM domain containing preferred translocation partner in lipoma
ADXCRSS.Hs#S1907985_at	2908	NR3C1	Nuclear receptor subfamily 3, group C, member 1 (glucocorticoid receptor)
ADXCRSS.Hs#S1910599_at	4026	LPP	LIM domain containing preferred translocation partner in lipoma
ADXCRSS.Hs#S1907164_at	55700	MAP7D1	MAP7 domain containing 1
ADXCRSS.Hs#S524340_at	8613	PPAP2B	Phosphatidic acid phosphatase type 2B
ADXCRSS.Hs#S2732925_at	9448	MAP4K4	Mitogen-activated protein kinase kinase kinase kinase 4
ADXCRSS.Hs#S2979568_at	5796	PTPRK	Protein tyrosine phosphatase, receptor type, K
ADXCRSS.Hs#S2984172_at	388743	CAPN8	Calpain 8
ADXCRSS.Hs#S2984613_at	9448	MAP4K4	Mitogen-activated protein kinase kinase kinase kinase 4
ADXCRSS.Hs#S2984613_x_at	9448	MAP4K4	Mitogen-activated protein kinase kinase kinase kinase 4
ADXCRSS.Hs#S2984275_at	4026	LPP	LIM domain containing preferred translocation partner in lipoma
ADXCRSS.Hs#S794492_at	5796	PTPRK	Protein tyrosine phosphatase, receptor type, K
ADXCRSS.Hs#S3747393_at	2273	FHL1	Four and a half LIM domains 1
ADXCRSS.Hs#S3748577_at	4026	LPP	LIM domain containing preferred translocation partner in lipoma
ADXCRSS.Hs#S3748482_at	87	ACTN1	Actinin, alpha 1
ADXCRSS.Hs#S3735513_at	4750	NEK1	NIMA (never in mitosis gene a)-related kinase 1
ADXCRSS.Hs#S3898008_at	NA	NA	
ADXCRSS.Hs#S821188_at	2908	NR3C1	Nuclear receptor subfamily 3, group C, member 1 (glucocorticoid receptor)
ADXCRIH.1382.C1_at	824	CAPN2	Calpain 2, (m/II) large subunit
ADXCRIH.1382.C2_s_at	824	CAPN2	Calpain 2, (m/II) large subunit
ADXCRIH.1441.C1_at	960	CD44	CD44 molecule (Indian blood group)
ADXCRIH.1441.C1_s_at	960	CD44	CD44 molecule (Indian blood group)
ADXCRIH.1441.C2_s_at	960	CD44	CD44 molecule (Indian blood group)
ADXCRIH.1926.C1_at	2274	FHL2	Four and a half LIM domains 2
ADXCRIH.1926.C1_s_at	2274	FHL2	Four and a half LIM domains 2
ADXCRIH.1926.C2_at	2274	FHL2	Four and a half LIM domains 2
ADXCRIH.1926.C2_s_at	2274	FHL2	Four and a half LIM domains 2
ADXCRPD.3100.C1_s_at	6709	SPTAN1	Spectrin, alpha, non-erythrocytic 1 (alpha-fodrin)
ADXCRPD.5728.C2_at	493	ATP2B4	ATPase, Ca++ transporting, plasma membrane 4
ADXCRPD.5728.C2_x_at	493	ATP2B4	ATPase, Ca++ transporting, plasma membrane 4
ADXCRPD.9848.C1_at	NA	NA	
ADXCRPD.9848.C1_s_at	6709	SPTAN1	Spectrin, alpha, non-erythrocytic 1 (alpha-fodrin)
ADXCRPD.9848.C2_at	6709	SPTAN1	Spectrin, alpha, non-erythrocytic 1 (alpha-fodrin)
ADXCRAD_BX404438_s_at	4907	NT5E	5'-nucleotidase, ecto (CD73)
ADXCRAD_BP319713_at	9706	ULK2	Unc-51-like kinase 2 (C. elegans)
ADXCRAD_BP319713_x_at	9706	ULK2	Unc-51-like kinase 2 (C. elegans)
ADXCRAD_BU508110_at	9444	QKI	Quaking homolog, KH domain RNA binding (mouse)
ADXCRAD_BU508110_x_at	9444	QKI	Quaking homolog, KH domain RNA binding (mouse)
ADXCRAD_CV026388_at	960	CD44	CD44 molecule (Indian blood group)
ADXCRAD_CV026388_x_at	960	CD44	CD44 molecule (Indian blood group)
ADXCRAD_AL545542_at	284119	PTRF	Polymerase I and transcript release factor
ADXCRAD_AL545542_x_at	284119	PTRF	Polymerase I and transcript release factor
ADXCRAD_BF855173_at	989	SEPT7	Septin 7
ADXCRAD_CD653165_at	NA	NA	
ADXCRAD_BP228866_s_at	9448	MAP4K4	Mitogen-activated protein kinase kinase kinase kinase 4
ADXCRAD_BP380290_s_at	813	CALU	Calumenin
ADXCRAD_CN332899_at	2316	FLNA	Filamin A, alpha (actin binding protein 280)
ADXCRAD_CN332899_x_at	2316	FLNA	Filamin A, alpha (actin binding protein 280)
ADXCRAD_CX784253_s_at	966	CD59	CD59 molecule, complement regulatory protein
ADXCRAD_CN431378_s_at	966	CD59	CD59 molecule, complement regulatory protein
ADXCRAD_BP343697_s_at	966	CD59	CD59 molecule, complement regulatory protein
ADXCRAD_CX872156_s_at	2273	FHL1	Four and a half LIM domains 1
ADXCRAD_CD110028_at	NA	NA	
ADXCRAD_AV732105_at	NA	NA	
ADXCRAD_AV732105_s_at	4082	MARCKS	Myristoylated alanine-rich protein kinase C substrate
ADXCRAD_CA392568_at	2908	NR3C1	Nuclear receptor subfamily 3, group C, member 1 (glucocorticoid receptor)
ADXCRAD_BQ433598_at	2908	NR3C1	Nuclear receptor subfamily 3, group C, member 1 (glucocorticoid receptor)
ADXCRAD_CX783483_at	28951	TRIB2	Tribbles homolog 2 (Drosophila)
ADXCRAD_CX783483_s_at	28951	TRIB2	Tribbles homolog 2 (Drosophila)
ADXCRAD_CD101810_at	NA	NA	
ADXCRAD_CD101810_x_at	51232	CRIM1	Cysteine rich transmembrane BMP regulator 1 (chordin-like)
ADXCRAD_AA234664_at	9263	STK17A	Serine/threonine kinase 17a
ADXCRAD_BM456598_at	4026	LPP	LIM domain containing preferred translocation partner in lipoma
ADXCRAD_BM456598_x_at	4026	LPP	LIM domain containing preferred translocation partner in lipoma
ADXCRAD_CF619371_at	389058	SP5	Sp5 transcription factor
ADXCRAD_CK818454_s_at	9706	ULK2	Unc-51-like kinase 2 (C. elegans)
ADXCRAD_BU941055_at	2152	F3	Coagulation factor III (thromboplastin, tissue factor)
ADXCRAD_BU941055_x_at	2152	F3	Coagulation factor III (thromboplastin, tissue factor)
ADXCRAD_BP233968_at	960	CD44	CD44 molecule (Indian blood group)
ADXCRAD_AV662263_s_at	3572	IL6ST	Interleukin 6 signal transducer (gp130, oncostatin M receptor)

ADXCRAD_BP254636_s_at	1112	FOXN3	Forkhead box N3
ADXCRAD_BX099460_at	439921	MXRA7	Matrix-remodelling associated 7
ADXCRAD_BM704188_at	4907	NT5E	5'-nucleotidase, ecto (CD73)
ADXCRAD_AW959541_s_at	55422	ZNF331	Zinc finger protein 331
ADXCRAD_AI651806_s_at	51232	CRIM1	Cysteine rich transmembrane BMP regulator 1 (chordin-like)
ADXCRAD_BG435621_at	NA	NA	
ADXCRAD_BM840380_s_at	960	CD44	CD44 molecule (Indian blood group)
ADXCRAD_BU501514_s_at	472	ATM	Ataxia telangiectasia mutated
ADXCRAD_CX871590_s_at	87	ACTN1	Actinin, alpha 1
ADXCRAD_BU186765_at	NA	NA	
ADXCRAD_BU186765_x_at	100506495	LIFR-AS1	
ADXCRAD_BM995629_s_at	1112	FOXN3	Forkhead box N3
ADXCRAD_CX872111_at	203068	TUBB	Tubulin, beta
ADXCRAD_CX872111_s_at	203068	TUBB	Tubulin, beta
ADXCRAD_CB956496_at	7026	NR2F2	Nuclear receptor subfamily 2, group F, member 2
ADXCRAD_CB956496_x_at	7026	NR2F2	Nuclear receptor subfamily 2, group F, member 2
ADXCRAD_AU132621_at	1063	CENPF	Centromere protein F, 350/400ka (mitosin)
ADXCRAD_BG258819_at	NA	NA	
ADXCRAD_BP361024_at	2273	FHL1	Four and a half LIM domains 1
ADXCRAD_BI833263_at	NA	NA	
ADXCRAD_AU280467_s_at	3572	IL6ST	Interleukin 6 signal transducer (gp130, oncostatin M receptor)
ADXCRAD_CN299966_at	493	ATP2B4	ATPase, Ca++ transporting, plasma membrane 4
ADXCRAD_CK002286_s_at	9444	QKI	Quaking homolog, KH domain RNA binding (mouse)
ADXCRAD_BM704951_at	7070	THY1	Thy-1 cell surface antigen
ADXCRAD_BM704951_x_at	7070	THY1	Thy-1 cell surface antigen
ADXCRAD_BP275873_s_at	23032	USP33	Ubiquitin specific peptidase 33
ADXCRAD_CA435088_s_at	6709	SPTAN1	Spectrin, alpha, non-erythrocytic 1 (alpha-fodrin)
ADXCRAD_CB268908_s_at	2534	FYN	FYN oncogene related to SRC, FGR, YES
ADXCRAD_AL552789_s_at	11031	RAB31	RAB31, member RAS oncogene family
ADXCRAD_CD299090_at	9200	PTPLA	Protein tyrosine phosphatase-like (proline instead of catalytic arginine), member A
ADXCRAD_CD299090_s_at	9200	PTPLA	Protein tyrosine phosphatase-like (proline instead of catalytic arginine), member A
ADXCRAD_CN347927_at	8682	PEA15	Phosphoprotein enriched in astrocytes 15
ADXCRAD_CD103045_at	4082	MARCKS	Myristoylated alanine-rich protein kinase C substrate
ADXCRAD_BI823993_s_at	3475	IFRD1	Interferon-related developmental regulator 1
ADXCRAD_BQ227453_s_at	9263	STK17A	Serine/threonine kinase 17a
ADXCRAD_CX867590_at	202243	CCDC125	Coiled-coil domain containing 125
ADXCRAD_CD677455_at	5813	PURA	Purine-rich element binding protein A
ADXCRAD_BM557039_at	NA	NA	
ADXCRAD_BQ223439_s_at	493	ATP2B4	ATPase, Ca++ transporting, plasma membrane 4
ADXCRAD_BP360257_at	22848	AAK1	AP2 associated kinase 1
ADXCRAD_BP360257_x_at	22848	AAK1	AP2 associated kinase 1
ADXCRAD_BQ720286_at	9444	QKI	Quaking homolog, KH domain RNA binding (mouse)
ADXCRAD_BQ720286_x_at	9444	QKI	Quaking homolog, KH domain RNA binding (mouse)
ADXCRAD_BQ436873_at	595	CCND1	Cyclin D1
ADXCRAD_BI869786_at	NA	NA	
ADXCRAD_CV804054_at	11004	KIF2C	Kinesin family member 2C
ADXCRAD_CV804054_s_at	11004	KIF2C	Kinesin family member 2C
ADXCRAD_BQ887381_at	112399	EGLN3	Egl nine homolog 3 (C. elegans)
ADXCRAD_BQ887381_s_at	3572	IL6ST	Interleukin 6 signal transducer (gp130, oncostatin M receptor)
ADXCRAD_BQ887381_x_at	3572	IL6ST	Interleukin 6 signal transducer (gp130, oncostatin M receptor)
ADXCRAD_CX762421_at	NA	NA	
ADXCRAD_CX762421_s_at	5621	PRNP	Prion protein (p27-30) (Creutzfeldt-Jakob disease, Gerstmann-Strausler-Scheinker syndrome, fatal familial insomnia)
ADXCRAD_BU178034_x_at	55700	MAP7D1	MAP7 domain containing 1
ADXCRAD_BG110060_at	NA	NA	
ADXCRAD_BU176917_at	55422	ZNF331	Zinc finger protein 331
ADXCRAD_NM_002228_s_at	3725	JUN	Jun oncogene
ADXCRAD_BI825165_at	NA	NA	
ADXCRAD_BQ647365_at	9448	MAP4K4	Mitogen-activated protein kinase kinase kinase kinase 4
ADXCRAD_CX866982_s_at	1009	CDH11	Cadherin 11, type 2, OB-cadherin (osteoblast)
ADXCRAD_BG611920_at	NA	NA	
ADXCRAD_BQ222360_at	NA	NA	
ADXCRAD_BQ222360_s_at	10049	DNAJB6	DnaJ (Hsp40) homolog, subfamily B, member 6
ADXCRAD_AJ712294_s_at	3725	JUN	Jun oncogene
ADXCRAD_CN295279_at	9444	QKI	Quaking homolog, KH domain RNA binding (mouse)
ADXCRAD_BM714793_at	87	ACTN1	Actinin, alpha 1
ADXCRAD_BM700086_s_at	9697	TRAM2	Translocation associated membrane protein 2
ADXCRAD_CN419328_s_at	26031	OSBPL3	Oxysterol binding protein-like 3
ADXCRAD_C16716_s_at	51232	CRIM1	Cysteine rich transmembrane BMP regulator 1 (chordin-like)
ADXCRAD_BP321052_s_at	51474	LIMA1	LIM domain and actin binding 1
ADXCRAD_NM_014683_at	9706	ULK2	Unc-51-like kinase 2 (C. elegans)
ADXCRAD_BC015940_at	4907	NT5E	5'-nucleotidase, ecto (CD73)
ADXCRAD_AL833171_at	4026	LPP	LIM domain containing preferred translocation partner in lipoma
ADXCRAD_BC016339_s_at	51232	CRIM1	Cysteine rich transmembrane BMP regulator 1 (chordin-like)
ADXCRAD_AW183478_at	9263	STK17A	Serine/threonine kinase 17a
ADXCRAD_AI831470_at	641977	SEPT7P2	
ADXCRAD_N29457_at	91687	CENPL	Centromere protein L
ADXCRAD_AI934556_at	2908	NR3C1	Nuclear receptor subfamily 3, group C, member 1 (glucocorticoid receptor)
ADXCRAD_M95178_at	5054	SERPINE1	Serpin peptidase inhibitor, clade E (nexin, plasminogen activator inhibitor type 1), member 1
ADXCRAD_M95178_x_at	5054	SERPINE1	Serpin peptidase inhibitor, clade E (nexin, plasminogen activator inhibitor type 1), member 1
ADXCRAD_AK023851_s_at	824	CAPN2	Calpain 2, (m/II) large subunit
ADXCRAD_AI282511_s_at	63826	SRR	Serine racemase
ADXCRAD_AA935633_at	9444	QKI	Quaking homolog, KH domain RNA binding (mouse)
ADXCRAD_AI079544_at	4026	LPP	LIM domain containing preferred translocation partner in lipoma
ADXCRAD_AI079544_s_at	4026	LPP	LIM domain containing preferred translocation partner in lipoma

ADXCRAD_N39126_at	989	SEPT7	Septin 7
ADXCRAD_N39126_s_at	989	SEPT7	Septin 7
ADXCRAD_AU145587_at	5796	PTPRK	Protein tyrosine phosphatase, receptor type, K
ADXCRAD_R41907_at	9444	QKI	Quaking homolog, KH domain RNA binding (mouse)
ADXCRAD_AA642418_s_at	51232	CRIM1	Cysteine rich transmembrane BMP regulator 1 (chordin-like)
