# Supplementary material for: Cell Line Derived 5-FU and Irinotecan Drug-Sensitivity Profiles Evaluated in Adjuvant Colon Cancer Trial Data
Source: PLoS One. 2016 May 12;11(5):e0155123. doi: 10.1371/journal.pone.0155123 (PMC4865183; doi:10.1371/journal.pone.0155123)
Supplement: S3 Table — Part A. Baseline demographics for the patients from the PETACC-3 study. The table provides an overview of the distribution of all patients in the PETACC-3 study according to the major clinicopathological variables. Some variables were only assessed in the profiled samples. This information is provided in italics. For example, the KRAS mutation status was not assessed in most of the samples (1395 missing values). Hence, the information that only 24% of the samples are wild-type is somewhat misleading: In italics, one can see that among the assessed samples, 60.4% were found to be wild-type and 39.4% to be KRAS mutated. Part B. Baseline demographics for the subset of stage III patients from the PETACC-3 study selected for the present analysis. The table provides an overview of the distribution of the PETACC-3 patients used in the present analysis according to the major clinicopathological variables. The information provided in italics shows the actual percentages among all samples without missing values. For example, the KRAS mutation status was not assessed in 40 samples (6.3% of the patients). Hence, the information that only 55.7% of the samples are wild-type can be misleading. In italics, one can see that actually 59.4% of the assessed samples were found to be wild-type and 40.6% to be KRAS mutated. Part C. Baseline demographics for the stage II CC patients from Kennedy et al [21]. The table provides an overview of the distribution of all (stage II) patients in the study published in Kennedy et al [21] according to the major clinicopathological variables. (PDF) [file pone.0155123.s005.pdf]

S3 Table part A

**Variable**	**all**	**FOLFIRI**	**5-FU/FA**
#samples	2315	1158	1157
age in years, median (range)	60 (18 , 76)	60 (18 , 76)	60 (21 , 76)
sex, n(%)			
male	1263 (54.6)	625 (54)	638 (55.1)
female	1052 (45.4)	533 (46)	519 (44.9)
site, n(%)			
left	1422 (61.4)	711 (61.4)	711 (61.5)
right	893 (38.6)	447 (38.6)	446 (38.5)
tstage, n(%)			
T12	196 (8.5)	95 (8.2)	101 (8.7)
T3	1766 (76.3)	865 (74.7)	901 (77.9)
T4	351 (15.2)	196 (16.9)	155 (13.4)
nstage, n(%)			
N1	1496 (64.6)	739 (63.8)	757 (65.4)
N2	819 (35.4)	419 (36.2)	400 (34.6)
grade, n(%)			
G-12	877 (37.9)	453 (39.1)	424 (36.6)
	877 (88.1)	*453 (88.8)*	*424 (87.2)*
G-34	119 (5.1)	57 (4.9)	62 (5.4)
	119 (11.9)	*57 (11.2)*	*62 (12.8)*
missing	1319 (57)	648 (56)	671 (58)
BRAF status, n(%)			
wild type	848 (36.6)	436 (37.7)	412 (35.6)
	848 (91.6)	*436 (92.2)*	*412 (90.9)*
mutant	78 (3.4)	37 (3.2)	41 (3.5)
	78 (8.4)	*37 (7.8)*	*41 (9.1)*
missing	1389 (60)	685 (59.2)	704 (60.8)
KRAS status, n(%)			
wild type	556 (24)	290 (25)	266 (23)
	556 (60.4)	*290 (62)*	*266 (58.8)*
mutant	364 (15.7)	178 (15.4)	186 (16.1)
	364 (39.6)	*178 (38)*	*186 (41.2)*
missing	1395 (60.3)	690 (59.6)	705 (60.9)
MSI status, n(%)			
MSS	772 (33.3)	392 (33.9)	380 (32.8)
	772 (87.9)	*392 (88.7)*	*380 (87.2)*
MSI-H	106 (4.6)	50 (4.3)	56 (4.8)
	106 (12.1)	*50 (11.3)*	*56 (12.8)*
missing	1437 (62.1)	716 (61.8)	721 (62.3)
RFS events, n(%)	947 (40.9)	451 (38.9)	496 (42.9)
OS events, n(%)	683 (29.5)	325 (28.1)	358 (30.9)

S3 Table part B

**Variable**	**all**	**FOLFIRI**	**5-FU/FA**
#samples	636	329	307
age in years, median (range)	61 (21 , 76)	62 (21 , 75)	60 (26 , 76)
sex, n(%)			
male	378 (59.4)	187 (56.8)	191 (62.2)
female	258 (40.6)	142 (43.2)	116 (37.8)
site, n(%)			
left	400 (62.9)	203 (61.7)	197 (64.2)
right	236 (37.1)	126 (38.3)	110 (35.8)
tstage, n(%)			
T12	54 (8.5)	27 (8.2)	27 (8.8)
T3	483 (75.9)	245 (74.5)	238 (77.5)
T4	99 (15.6)	57 (17.3)	42 (13.7)
nstage, n(%)			
N1	418 (65.7)	212 (64.4)	206 (67.1)
N2	218 (34.3)	117 (35.6)	101 (32.9)
grade, n(%)			
G-12	565 (88.8)	295 (89.7)	270 (87.9)
	565 (89.4)	*295 (90.5)*	*270 (88.2)*
G-34	67 (10.5)	31 (9.4)	36 (11.7)
	67 (10.6)	*31 (9.5)*	*36 (11.8)*
missing	4 (0.6)	3 (0.9)	1 (0.3)
BRAF status, n(%)			
wild type	558 (87.7)	289 (87.8)	269 (87.6)
	558 (93.3)	*289 (93.5)*	*269 (93.1)*
mutant	40 (6.3)	20 (6.1)	20 (6.5)
	40 (6.7)	*20 (6.5)*	*20 (6.9)*
missing	38 (6)	20 (6.1)	18 (5.9)
KRAS status, n(%)			
wild type	354 (55.7)	185 (56.2)	169 (55)
	354 (59.4)	*185 (60.3)*	*169 (58.5)*
mutant	242 (38.1)	122 (37.1)	120 (39.1)
	242 (40.6)	*122 (39.7)*	*120 (41.5)*
missing	40 (6.3)	22 (6.7)	18 (5.9)
MSI status, n(%)			
MSS	512 (80.5)	263 (79.9)	249 (81.1)
	512 (90.3)	*263 (91)*	*249 (89.6)*
MSI-H	55 (8.6)	26 (7.9)	29 (9.4)
	55 (9.7)	*26 (9)*	*29 (10.4)*
missing	69 (10.8)	40 (12.2)	29 (9.4)
RFS events, n(%)	239 (37.6)	117 (35.6)	122 (39.7)
OS events, n(%)	179 (28.1)	84 (25.5)	95 (30.9)

S3 Table part C

**Variable**	samples
#samples	359
age in years, median (range)	71 (45 , 95)
sex, n(%)	
male	175 (48.7)
female	184 (51.3)
site, n(%)	
left	41 (11.4)
right	167 (46.5)
missing	151 (42.1)
tstage, n(%)	
T3	307 (85.5)
T4	52 (14.5)
RFS events, n(%)	137 (38.2)
OS events, n(%)	137 (38.2)
Follow-up time in months, median (range)	67 (3 , 212)
