# Supplementary material for: Cell Line Derived 5-FU and Irinotecan Drug-Sensitivity Profiles Evaluated in Adjuvant Colon Cancer Trial Data
Source: PLoS One. 2016 May 12;11(5):e0155123. doi: 10.1371/journal.pone.0155123 (PMC4865183; doi:10.1371/journal.pone.0155123)
Supplement: S4 Table — Part A. Association between the irinotecan profile score and clinicopathological parameters for the PETACC-3 subpopulation. We tested for association between the irinotecan profile score and the major clinicopathological parameters in the clinical data. It is seen that site, grade and MSI has a significant association with the irinotecan profile score in the PETACC-3 subpopulation in the simple and in the multiple regression models. Detailed results are provided in the table below. The first two columns relate to the results from a multivariable regression model. The last two columns relate to the results of each variable being tested in a simple (single explanatory variable) model. The estimates for the intercepts are not reported. Part B. Association between RFS and the irinotecan profile score in the subpopulation of the PETACC-3 study. The first three columns relate to results from a multivariable Cox Proportional Hazards model. The last three columns to the results of each variable was tested in a simple (single explanatory variable) Cox Proportional Hazards model. The irinotecan profile score was not statistically significantly associated with RFS. The estimates for the other variables are in line with results obtained in former analyses on the PETACC-3 data. Part C. Association between OS and the irinotecan profile score in the subpopulation of the PETACC-3 study. The first three columns relate to results from a multivariable Cox Proportional Hazards model. The last three columns to the results of each variable was tested in a simple (single explanatory variable) Cox Proportional Hazards model. The irinotecan profile score was not statistically significantly associated with OS. Part D. Interaction between the irinotecan profile score and the treatment group, RFS and OS. In order to test for a potential interaction between the profile score and the treatment group, we fitted models for RFS and for OS including an interaction term (outcome ~ treatment_group + p [file pone.0155123.s006.pdf]

S4 Table part A

	coef_multi	pval_multi	coef_sing	pval_sing
trt_grp (FOLFIRI vs 5-FU/FA)	-0.05	0.42672	-0.02	0.71085
age (in years)	0.00	0.95150	-0.00	0.27997
sex (female vs male)	-0.00	0.95795	-0.01	0.87124
site (right vs left)	0.19	0.00598	0.17	0.00814
tstage (T12 vs T3)	-0.15	0.16160	-0.17	0.13754
tstage (T4 vs T3)	0.06	0.49641	0.05	0.55842
nstage (N2 vs N1)	0.09	0.22348	0.12	0.06946
grade (G-34 vs G-12)	0.09	0.45266	0.22	0.02764
BRAF (mut vs wt)	-0.36	0.00769	-0.08	0.55136
KRAS (mut vs wt)	-0.10	0.13938	-0.07	0.25772
MSI (MSI-H vs MSS)	0.40	0.00076	0.47	0.00001

Full model: n = 558, 78 observations deleted due to missingness

S4 Table part B

	HR_multi	CI_multi	pval_multi	HR_sing	CI_sing	pval_sing
IrinoPred (IQR scaled)	0.93	(0.77, 1.12)	0.44994	0.94	(0.8, 1.11)	0.44636
trt_grp (FOLFIRI vs 5-FU/FA)	0.82	(0.63, 1.08)	0.16541	0.87	(0.68, 1.12)	0.29253
age (in years)	1.00	(0.99, 1.01)	0.95855	1.00	(0.99, 1.01)	0.73414
sex (female vs male)	0.99	(0.74, 1.31)	0.92017	0.91	(0.7, 1.18)	0.46790
site (right vs left)	1.26	(0.94, 1.7)	0.12368	1.10	(0.85, 1.43)	0.47551
tstage (T12 vs T3)	0.50	(0.26, 1)	0.04919	0.41	(0.21, 0.8)	0.00894
tstage (T4 vs T3)	1.81	(1.3, 2.51)	0.00044	1.86	(1.38, 2.52)	0.00005
nstage (N2 vs N1)	2.04	(1.54, 2.71)	0.00000	2.21	(1.71, 2.85)	0.00000
grade (G-34 vs G-12)	1.57	(0.99, 2.48)	0.05488	1.57	(1.09, 2.27)	0.01570
BRAF (mut vs wt)	1.14	(0.64, 2.02)	0.65877	1.16	(0.7, 1.94)	0.55788
KRAS (mut vs wt)	1.45	(1.09, 1.95)	0.01223	1.29	(0.99, 1.68)	0.05835
MSI (MSI-H vs MSS)	0.45	(0.25, 0.81)	0.00730	0.60	(0.35, 1.03)	0.06384

Full model: n = 558, number of events = 212, 78 observations deleted due to missingness

S4 Table part C

	HR_multi	CI_multi	pval_multi	HR_sing	CI_sing	pval_sing
IrinoPred (IQR scaled)	0.96	(0.78, 1.18)	0.69570	0.97	(0.8, 1.17)	0.73924
trt_grp (FOLFIRI vs 5-FU/FA)	0.79	(0.57, 1.08)	0.14300	0.83	(0.62, 1.11)	0.20021
age (in years)	1.00	(0.98, 1.02)	0.99646	1.01	(0.99, 1.02)	0.35061
sex (female vs male)	0.88	(0.63, 1.22)	0.44032	0.80	(0.59, 1.09)	0.16094
site (right vs left)	1.50	(1.07, 2.11)	0.02016	1.41	(1.05, 1.89)	0.02304
tstage (T12 vs T3)	0.51	(0.22, 1.17)	0.11096	0.38	(0.17, 0.85)	0.01928
tstage (T4 vs T3)	2.20	(1.51, 3.19)	0.00004	2.04	(1.45, 2.86)	0.00004
nstage (N2 vs N1)	2.05	(1.48, 2.85)	0.00002	2.40	(1.79, 3.21)	0.00000
grade (G-34 vs G-12)	1.76	(1.04, 2.97)	0.03533	1.91	(1.28, 2.87)	0.00161
BRAF (mut vs wt)	1.85	(1.01, 3.4)	0.04697	1.76	(1.05, 2.94)	0.03189
KRAS (mut vs wt)	1.85	(1.31, 2.61)	0.00052	1.47	(1.09, 1.99)	0.01280
MSI (MSI-H vs MSS)	0.41	(0.21, 0.8)	0.00879	0.66	(0.36, 1.22)	0.19001

Full model: n = 558, number of events = 159, 78 observations deleted due to missingness

S4 Table part D

RFS

	HR	CI	pval
IrinoPred (IQR scaled)	0.95	(0.77, 1.18)	0.66321
trt_grp (FFIRI vs 5-F/F)	0.97	(0.41, 2.3)	0.95133
IrinoPred:trt_grp	0.96	(0.69, 1.33)	0.78965

n = 636, number of events = 239, 0 observations deleted due to missingness

OS

	HR	CI	pval
IrinoPred (IQR scaled)	0.95	(0.75, 1.21)	0.68884
trt_grp (FFIRI vs 5-F/F)	0.75	(0.28, 2.03)	0.57094
IrinoPred:trt_grp	1.04	(0.71, 1.52)	0.84470

n = 636, number of events = 179, 0 observations deleted due to missingness
