# Supplementary material for: Cell Line Derived 5-FU and Irinotecan Drug-Sensitivity Profiles Evaluated in Adjuvant Colon Cancer Trial Data
Source: PLoS One. 2016 May 12;11(5):e0155123. doi: 10.1371/journal.pone.0155123 (PMC4865183; doi:10.1371/journal.pone.0155123)
Supplement: S5 Table — Part A. Association between the 5-FU profile score and clinicopathological parameters for the PETACC-3 subpopulation. We tested for association between the 5-FU profile score and the major clinicopathological parameters in the clinical data. It is seen that T stage has a significant association with the 5-FU profile in the PETACC-3 subpopulation in the simple and in the multiple regression models. However, the effect is only statistically significant when comparing T1-2 (smallest group) to T3 (largest group). Detailed results are provided in the table below. The first two columns relate to the results from a multivariable regression model. The last two columns relate to the results of each variable being tested in a simple (single explanatory variable) model. The estimates for the intercepts are not reported. Part B. Association between OS and the 5-FU profile score in the subpopulation of the PETACC-3 study. The first three columns relate to results from a multivariable Cox Proportional Hazards model. The last three columns relate to the results of each variable being tested in a simple (single explanatory variable) Cox Proportional Hazards model. The 5-FU profile score is statistically significantly associated with OS, even when correcting for other covariates. Part C. Interaction between the 5-FU profile score and the treatment group, RFS and OS. In order to test for a potential interaction between the profile score and the treatment group, we fitted models for RFS and for OS including an interaction term (outcome ~ treatment_group + profile_score + treatment_group:profile_score). For the 5-FU profile, only the HR of the profile score was statistically significantly different from 1 after adjustment for the other explanatory variables in the model. The treatment group and the interaction term did not have a HR statistically significantly different from 1. (PDF) [file pone.0155123.s007.pdf]

S5 Table part A

	coef_multi	pval_multi	coef_sing	pval_sing
trt_grp (FOLFIRI vs 5-FU/FA)	0.04	0.61198	0.01	0.82616
age (in years)	0.00	0.22449	0.01	0.09206
sex (female vs male)	0.17	0.02547	0.14	0.03957
site (right vs left)	-0.15	0.05357	-0.14	0.04086
tstage (T12 vs T3)	0.45	0.00036	0.47	0.00013
tstage (T4 vs T3)	-0.18	0.07416	-0.14	0.13371
nstage (N2 vs N1)	-0.08	0.29567	-0.12	0.10243
grade (G-34 vs G-12)	0.11	0.40702	-0.07	0.52667
BRAF (mut vs wt)	-0.10	0.53326	-0.21	0.13849
KRAS (mut vs wt)	0.03	0.72373	0.05	0.50774
MSI (MSI-H vs MSS)	-0.04	0.78470	-0.16	0.19625

Full model: n = 558, 78 observations deleted due to missingness

S5 Table part B

	HR_multi	CI_multi	pval_multi	HR_sing	CI_sing	pval_sing
FU5Pred (IQR scaled)	0.69	(0.58, 0.82)	0.00002	0.64	(0.55, 0.75)	0.00000
trt_grp (FOLFIRI vs 5-FU/FA)	0.83	(0.61, 1.14)	0.25740	0.83	(0.62, 1.11)	0.20021
age (in years)	1.00	(0.99, 1.02)	0.79197	1.01	(0.99, 1.02)	0.35061
sex (female vs male)	0.93	(0.67, 1.3)	0.67739	0.80	(0.59, 1.09)	0.16094
site (right vs left)	1.38	(0.98, 1.94)	0.06248	1.41	(1.05, 1.89)	0.02304
tstage (T12 vs T3)	0.62	(0.27, 1.44)	0.26566	0.38	(0.17, 0.85)	0.01928
tstage (T4 vs T3)	2.03	(1.4, 2.95)	0.00021	2.04	(1.45, 2.86)	0.00004
nstage (N2 vs N1)	2.01	(1.45, 2.78)	0.00003	2.40	(1.79, 3.21)	0.00000
grade (G-34 vs G-12)	1.81	(1.07, 3.07)	0.02789	1.91	(1.28, 2.87)	0.00161
BRAF (mut vs wt)	1.90	(1.04, 3.45)	0.03590	1.76	(1.05, 2.94)	0.03189
KRAS (mut vs wt)	1.89	(1.34, 2.68)	0.00030	1.47	(1.09, 1.99)	0.01280
MSI (MSI-H vs MSS)	0.42	(0.22, 0.81)	0.01025	0.66	(0.36, 1.22)	0.19001

Full model: n = 558, number of events = 159, 78 observations deleted due to missingness

S5 Table part C

RFS

	HR	CI	pval
FU5Pred (IQR scaled)	0.72	(0.59, 0.87)	0.00089
trt_grp (FFIRI vs 5-F/F)	1.14	(0.46, 2.83)	0.77659
FU5Pred:trt_grp	0.92	(0.7, 1.22)	0.57421

n = 636, number of events = 239, 0 observations deleted due to missingness

OS

	HR	CI	pval
FU5Pred (IQR scaled)	0.65	(0.52, 0.8)	0.00009
trt_grp (FFIRI vs 5-F/F)	0.84	(0.31, 2.29)	0.73239
FU5Pred:trt_grp	1.00	(0.73, 1.36)	0.98347

n = 636, number of events = 179, 0 observations deleted due to missingness
