# Supplementary material for: Cell Line Derived 5-FU and Irinotecan Drug-Sensitivity Profiles Evaluated in Adjuvant Colon Cancer Trial Data
Source: PLoS One. 2016 May 12;11(5):e0155123. doi: 10.1371/journal.pone.0155123 (PMC4865183; doi:10.1371/journal.pone.0155123)
Supplement: S6 Table — Part A. Association between the 5-FU profile score and clinicopathological parameters for the samples from the Kennedy cohort. The first two columns relate to the results from a multivariable regression model. The last two columns relate to the results of each variable being tested in a simple (single explanatory variable) model. The estimates for the intercepts are not reported in the table below. The only statistically significant association found is with 'age'. Part B. Association between relapse free survival and the 5-FU profile score in the Kennedy cohort. The first three columns relate to results from a multivariable Cox Proportional Hazards model. The last three columns relate to the results of each variable being tested in a simple (single explanatory variable) Cox Proportional Hazards model. The 5-FU profile score was not statistically significantly associated with RFS. Part C. Association between overall survival and the 5-FU profile score in the Kennedy cohort. The first three columns relate to results from a multivariable Cox Proportional Hazards model. The last three columns relate to the results of each variable being tested in a simple (single explanatory variable) Cox Proportional Hazards model. The 5-FU profile score was not statistically significantly associated with OS. (PDF) [file pone.0155123.s008.pdf]

S6 Table part A

	coef_multi	pval_multi	coef_sing	pval_sing
age (in years)	0.01	0.04023	0.01	0.04160
sex (female vs male)	-0.05	0.60818	-0.03	0.70468
tstage (T4 vs T3)	-0.06	0.63600	-0.07	0.60295

Full model: n = 359, 0 observations deleted due to missingness

S6 Table part B

	HR_multi	CI_multi	pval_multi	HR_sing	CI_sing	pval_sing
FU5Pred (IQR scaled)	0.97	(0.8, 1.17)	0.74748	0.99	(0.82, 1.21)	0.93826
age (in years)	1.03	(1.01, 1.05)	0.00309	1.03	(1.01, 1.04)	0.00500
sex (female vs male)	0.87	(0.62, 1.22)	0.41205	0.93	(0.66, 1.3)	0.66338
tstage (T4 vs T3)	1.86	(1.24, 2.8)	0.00271	1.82	(1.21, 2.74)	0.00374

Full model: n = 359, number of events = 137, 0 observations deleted due to missingness

S6 Table part C

	HR_multi	CI_multi	pval_multi	HR_sing	CI_sing	pval_sing
FU5Pred (IQR scaled)	0.97	(0.8, 1.18)	0.76386	1.02	(0.84, 1.24)	0.85884
age (in years)	1.04	(1.02, 1.06)	0.00003	1.04	(1.02, 1.06)	0.00003
sex (female vs male)	0.98	(0.7, 1.38)	0.92341	1.09	(0.78, 1.52)	0.62849
tstage (T4 vs T3)	1.43	(0.91, 2.27)	0.12333	1.39	(0.88, 2.2)	0.15646

Full model: n = 359, number of events = 137, 0 observations deleted due to missingness
